# CodY Regulates Thiol Peroxidase Expression as Part of the Pneumococcal Defense Mechanism against H_2_O_2_ Stress

**DOI:** 10.3389/fcimb.2017.00210

**Published:** 2017-05-24

**Authors:** Barak Hajaj, Hasan Yesilkaya, Sulman Shafeeq, Xiangyun Zhi, Rachel Benisty, Shiran Tchalah, Oscar P. Kuipers, Nurith Porat

**Affiliations:** ^1^Pediatric Infectious Disease Unit, Department of Microbiology and Immunology, Faculty of Health Sciences, Soroka University Medical Center, Ben-Gurion University of the NegevBeer Sheva, Israel; ^2^Department of Infection, Immunity and Inflammation, University of LeicesterLeicester, United Kingdom; ^3^Department of Molecular Genetics, Groningen Biomolecular Sciences and Biotechnology Institute, University of GroningenGroningen, Netherlands

**Keywords:** *Streptococcus pneumoniae*, TpxD, CodY, global gene regulation, H_2_O_2_

## Abstract

*Streptococcus pneumoniae* is a facultative anaerobic pathogen. Although it maintains fermentative metabolism, during aerobic growth pneumococci produce high levels of H_2_O_2_, which can have adverse effects on cell viability and DNA, and influence pneumococcal interaction with its host. The pneumococcus is unusual in its dealing with toxic reactive oxygen species (ROS) in that it neither has catalase nor the global regulators of peroxide stress resistance. Previously, we identified pneumococcal thiol peroxidase (TpxD) as the key enzyme for enzymatic removal of H_2_O_2_, and showed that TpxD synthesis is up-regulated upon exposure to H_2_O_2_. This study aimed to reveal the mechanism controlling TpxD expression under H_2_O_2_ stress. We hypothesize that H_2_O_2_ activates a transcription factor which in turn up-regulates *tpxD* expression. Microarray analysis revealed a pneumococcal global transcriptional response to H_2_O_2_. Mutation of *tpxD* abolished H_2_O_2_-mediated response to high H_2_O_2_ levels, signifying the need for an active TpxD under oxidative stress conditions. Bioinformatic tools, applied to search for a transcription factor modulating *tpxD* expression, pointed toward CodY as a potential candidate. Indeed, a putative 15-bp consensus CodY binding site was found in the proximal region of *tpxD-*coding sequence. Binding of CodY to this site was confirmed by EMSA, and genetic engineering techniques demonstrated that this site is essential for TpxD up-regulation under H_2_O_2_ stress. Furthermore, *tpxD* expression was reduced in a Δ*codY* mutant. These data indicate that CodY is an activator of *tpxD* expression, triggering its up-regulation under H_2_O_2_ stress. In addition we show that H_2_O_2_ specifically oxidizes the 2 CodY cysteines. This oxidation may trigger a conformational change in CodY, resulting in enhanced binding to DNA. A schematic model illustrating the contribution of TpxD and CodY to pneumococcal global transcriptional response to H_2_O_2_ is proposed.

## Introduction

*Streptococcus pneumoniae* commonly causes severe infections such as pneumonia, meningitis, and septicemia. More than 2 million deaths per year are caused as a result of pneumococcal septicemia (O'Brien et al., 2009). The pneumococcus has the ability to colonize and invade into different tissue sites with distinct environmental conditions; however, the mechanisms enabling its survival in the host are poorly understood. One of the detriments of pneumococcal survival in the host is its exposure to toxic levels of reactive oxygen species (ROS) such as H_2_O_2_, produced by the host immune cells. Furthermore, during aerobic growth the pneumococcus produces extremely high levels of H_2_O_2_, up to 2 mM (Pericone et al., 2003; Taniai et al., 2008). H_2_O_2_ increases the mutation rate, leads to DNA damage and apoptosis in lung cells and has damaging effects on many macromolecular compounds (Pericone et al., 2002, 2003; Rai et al., 2015). On the other hand, peroxide production was suggested to provide a competitive advantage to the pneumococcus against other colonizing bacteria in the upper respiratory tract (Pericone et al., 2000). H_2_O_2_ plays a pivotal role in pneumococcal pathogenesis through its cytotoxicity to epithelial cells (Duane et al., 1993), and through induction of genes required for host inflammatory response (Loose et al., 2014). Therefore, it is important to understand how the pneumococcus responds to this toxic compound.

*S. pneumoniae* lacks the common proteins that have been shown to protect against oxidative stress in other bacterial species, such as the known H_2_O_2_ scavengers, catalase and NADH-peroxidase (Hoskins et al., 2001; Tettelin et al., 2001). Nonetheless, previous studies have shown that *S. pneumoniae* uses other key enzymes to defend itself against oxidative stress, such as superoxide dismutase (Yesilkaya et al., 2000), NADH oxidase (Auzat et al., 1999), and alkyl hydroperoxidase (Paterson et al., 2006).

We have previously shown that the pneumococcal surface adhesion D gene, *psaD*, encodes a functional thiol peroxidase, which scavenges directly H_2_O_2_, and hence renamed it as *tpxD* (Hajaj et al., 2012). *tpxD* expression and synthesis were significantly up-regulated under aerobic compared to anaerobic conditions, and in the presence of exogenously added (10–1,000 μM) H_2_O_2_. In addition, H_2_O_2_ removal by catalase resulted in a drop in *tpxD* expression and synthesis (Hajaj et al., 2012).

Studies in other bacteria have shown that the change in thiol peroxidase expression in response to H_2_O_2_ is regulated by two transcription factors (TFs), OxyR and PerR (Hoskins et al., 2001; Paget and Buttner, 2003; Veal et al., 2007; Chiang and Schellhorn, 2012; Mishra and Imlay, 2012), but *S. pneumoniae* lacks these two TFs. However, the pneumococcus has a regulator named CodY, which regulates various metabolic pathways and cellular processes. The DNA binding activity of CodY in *S. pneumoniae* is increased by branched chain amino acids [BCAA: isoleucine, leucine and valine (ILV)] (Brinsmade et al., 2010). Notably, pneumococcal CodY does not respond to GTP (Hendriksen et al., 2008), unlike *Bacillus subtilis, Staphylococcus aureus* and several other bacterial species (Han et al., 2016). Inactivation of *codY* in *S. pneumoniae* dramatically decreased adherence to nasopharyngeal cells, an essential stage for successful pneumococcal infection (Hendriksen et al., 2008). No significant differences in bacterial loads were observed with the pneumonia and sepsis model of infection (Hendriksen et al., 2008). Although CodY has also been implicated in regulation of pneumococcal oxidative stress resistance through its role in iron metabolism (Caymaris et al., 2010; Johnston et al., 2015), the extent of CodY-mediated regulation of oxidative resistance has not been studied in detail.

This study aimed to reveal the mechanism controlling TpxD expression under H_2_O_2_ stress. We hypothesize that H_2_O_2_ activates a transcription factor which in turn up-regulates *tpxD* expression. We present data showing that H_2_O_2_ induces a global transcriptional response in *S. pneumoniae* and that TpxD enables this response by limiting H_2_O_2_ levels. Furthermore, we identified a conserved CodY-binding site (AATCATTGGAAAATT) in the putative regulatory region of *tpxD*, and present mechanistic evidence that CodY serves as an activator of *tpxD*, thereby preventing toxic levels of H_2_O_2_.

## Materials and methods

### Bacterial strains and growth condition

*S. pneumoniae* serotype 2 strain, D39 (WT), and its isogenic mutants were used in this work. Routinely, pneumococci were grown anaerobically in closed, completely filled 20 ml test-tubes, in a water bath without shaking, at 37°C in Todd–Hewitt (TH) broth with 0.5% (w/v) yeast extract (THY) to an approximate OD_620_ of 0.25. Where specified, bacteria were grown in chemically defined medium (CDM) (Yesilkaya et al., 2009), with the exception that BCAA were omitted from the amino acids mixture, and added separately. D39 strains mutated in *tpxD* (Δ*tpxD*) and *brnQ* (Δ*brnQ*), were grown in medium supplemented with 100 μg ml^−1^ spectinomycin. The 6 Δ*tpxD* complemented strains 1–6 (Δ*tpxD*comp1-6; **Figure 2**), were grown in medium supplemented with 100 μg ml^−1^ spectinomycin and 250 μg ml^−1^ kanamycin. The Δ*codY* mutant (kindly donated by Dr. Calum Johnston, Laboratoire de Microbiologie et Genetique Moleculaires, France) was grown in medium supplemented with 20 μg ml^−1^ trimethoprim.

### Construction of genetically modified strains

The construction of Δ*tpxD* and its genetically complemented strain containing the entire *tpxD-*coding sequence *(SPD1464)* plus 83 bp upstream, was described previously (Hajaj et al., 2012). The *brnQ* gene disruption was achieved by allelic replacement mutagenesis as described previously (Song et al., 2005). Briefly, the upstream and downstream flanking regions of *brnQ* were amplified using the primer combinations LFSPD0546F/LFSPD0546R, for the upstream region, and RFSPD0546F/RFSPD0546R, for the downstream region, respectively (Table S1). Subsequently, using LFSPD0546F and RFSPD0546R primers, the flanking fragments were fused to the spectinomycin resistance marker, which had been amplified with specF and specR primers from pDL278 (Yesilkaya et al., 2000). The resulting fused fragments were purified and transformed into *S. pneumoniae* D39 as described before using competence stimulating peptide (Alloing et al., 1996). The transformants were selected on blood agar base (BAB) containing 5% (v/v) sheep blood supplemented with spectinomycin. The mutation was confirmed by PCR using different primer combinations, and DNA sequencing.

To construct the 6 genetically *cis*-complemented strains (Δ*tpxD*comp1-6), the chromosomal region containing the entire *tpxD*-coding sequence preceded by a variable length of the proximal upstream sequence harboring different parts of *tpxD* putative regulatory elements, was amplified using the appropriate primers (Table S1), which were designed to include *NcoI* or *BamHI* sites. The amplicons were purified (Qiagen), digested and cloned into similarly digested pCEP (Guiral et al., 2006). An aliquot of ligation mixture was transformed into *E. coli* BL21 (DE3), and kanamycin resistant transformants were analyzed for the presence of recombinant plasmid using primers, malF and pCEPR, that anneal to either side of the cloning site. Then the purified recombinant plasmid was transformed into Δ*tpxD* as described previously (Alloing et al., 1996). The successful construction of modified strains was then confirmed by PCR using malF and pCEPR primers, and DNA sequencing.

### Point mutation of TpxD catalytic cysteine^58^

The replacement of cys^58^ to serine in TpxD was done by splicing overlap extension (Song et al., 2005). For this, the region surrounding cys^58^ was amplified using malF and TPX-S1, and pCEPR and TPX-S2 primers (Table S1). The amplicons were then fused together using malF and pCEPR primers. The fused products were digested with *BamHI* and *NcoI*, and ligated into the same sites of pCEP. The replacement of nucleotide sequence was confirmed by sequencing using malF and pCEPR primers, and the recombinant plasmid was transformed into Δ*tpxD* as before. The resulting strain was designated as TpxDC58S.

### Relative gene expression

Bacteria were grown anaerobically at 37°C in THY medium to OD_620_ = 0.25 and then challenged with 100, 250 or 1000 μM H_2_O_2_ for 40 min while maintaining anaerobic conditions. Control cultures were incubated with Double-distilled water instead of H_2_O_2_. Viability of WT and Δ*tpxD* bacteria, challenged with 1 mM H_2_O_2_, was confirmed by colony forming unit counts, as previously described (Hajaj et al., 2012). Experiments were repeated at least twice, each with two biological replicates. RNA was prepared by using MasterPure^TM^ RNA purification kit (Epicentre®, USA). RNA was treated with DNase I according to the above kit protocol. cDNA was synthesized with Verso^TM^ cDNA kit (ABgene, UK). Real-time PCR reactions contained Absolute^TM^ blue QPCR SYBR mix ROX (ABgene, UK), and the expression of individual genes was determined using gene specific primers (Table S1). The transcription level of target genes was normalized to *gyrA*. The results were analyzed by the comparative C_T_ method (Livak and Schmittgen, 2001).

### DNA microarray analysis

D39 and its isogenic Δ*tpxD* were grown anaerobically at 37°C in THY medium to OD_620_ = 0.25 and then challenged with 1 mM H_2_O_2_ for 40 min while maintaining anaerobic conditions. Control cultures were incubated with Double-distilled water instead of H_2_O_2_. All other procedures regarding DNA microarray experiments (RNA isolation, RNA quality test, cDNA synthesis and labeling) were performed as described (Shafeeq et al., 2011). Microarray slide images were scanned using GenPix Pro 6.1 (MSD analytical technologies). Processing and normalization (LOWESS spotpin-based) of slides was done with the in-house developed MicroPrep software. DNA microarray data were obtained from three independent biological replicates hybridized to glass slides with a dye swap. Expression ratios were calculated from the measurements of at least seven spots. Differential expression tests were performed on expression ratios with a local copy of the Cyber-T implementation of a variant of the *t*-test. False discovery rates were calculated as described (van Hijum et al., 2005). A gene with *p*-value of < 0.005 and a fold change cut-off of 1.8 was considered differential expressed. We have used the Cyber-T server to analyze our Microarray data. Therefore, the *p*-values mentioned in this study (Supplementary Material) should be considered as Bayes corrected *p*-values.

Microarray data have been submitted to Gene Expression Omnibus (GEO) database under the accession number GSE65157.

### Expression, purification, and antibody production of TpxD

The coding sequence of *tpxD* was amplified by PCR with tpx-for and tpx-rev primers (Table S1) from *S. pneumoniae* D39 and cloned into pRSETc vector (Invitrogen) between XhoI and KpnI sites. *E. coli* BL21 cells harboring the constructed plasmid were grown in Luria-Bertani broth supplemented with ampicillin (100 μg/ml) for 24 h. Cells were harvested by centrifugation and stored at −70 °C. The pellet was suspended in lysis buffer [50 mM Tris pH 8, 100 mM phenylmethylsulfonyl fluoride (PMSF)], disintegrated by sonication, and centrifuged at 4000 × g for 1 h. Proteins in the supernatant were loaded onto a Ni-NTA column (Adar biotec, Israel), and incubated for 1 h at 4°C. The column was then washed with 10 mM imidazole, and the recombinant protein eluted from the column with 100 mM imidazole. The eluted protein was run on SDS-PAGE and a band which was of the predicted molecular weight of TpxD was visualized. Polyclonal antibodies against TpxD were custom made by Sigma Aldrich, Israel, using the purified recombinant TpxD.

### SDS-PAGE and western blotting

Pneumococci were lysed and subjected to SDS-PAGE. Separated proteins were electroblotted onto 0.45 μm nitrocellulose membrane (Bio-Rad), and probed with the polyclonal antibodies against TpxD. Antigen complexes were detected using Peroxidase Affinity Pure Goat anti rabbit IgG (Jackson ImmunoResearch) and visualized with SuperSignal West Pico Chemiluminescent substrate (Pierce). Densitometry was performed using ImageJ software (Schneider et al., 2012).

### Computational analysis of *tpxD* promoter region and regulatory sites

The sequence of *tpxD* upstream region in D39 was extracted from the National Center for Biotechnology Information (NCBI) database using the following entries: *tpxD* sequence gene ID: 4441865. *Streptococcus pneumoniae* D39 strain sequence ID: NC_008533.1. Promoter elements were predicted using the Softberry BPROM algorithm of bacterial promoters (http://www.softberry.com/berry.phtml?topic=bprom&group=programs&subgroup=gfindb). Known CodY-regulated genes in *S. pneumoniae* were retrieved from the RegPrecise database (Novichkov et al., 2010), and their CodY binding site aligned with the upstream sequence of *tpxD*, using ClustalW in BioEdit 7.0.9 (Hall, 1999). CodY binding motif was visualized using WebLogo 3.2 (Lewis et al., 2000).

### Cloning, expression and purification of CodY

The coding sequence of *codY* was amplified by PCR from Genomic DNA from D39 strain with CodYF and CodYR primers (Table S1). The amplicons were cloned into plasmid pLEIC using In-fusion Cloning kit (Clontech), which allows ligation independent cloning method based on the annealing of complementary ends of a cloning insert and linearized cloning vector. The recombinant construct was transformed into Fusion-Blue competent *E. coli*, and transformants were plated on LA containing 100 μg/ml ampicillin. A transformant was selected for sequencing to rule out any mutation. The construct DNA then was transformed into *E. coli* BL21(DE3) (Novagen, United Kingdom) for recombinant protein expression. The strain with the recombinant plasmid was grown in Luria-Bertani broth containing ampicillin (100 μg/ml) for 16 h at 25°C, and the expression was induced with 0.5 mM Isopropyl β-D-1-thiogalactopyranoside. The pellet was collected by centrifugation, resuspended in lysis buffer (50 mM Tris pH 8, 100 mM PMSF), and subjected to sonication. The protein purification used immobilized metal affinity chromatography (IMAC) resin and non-denaturing conditions as instructed by the manufacturer (Clontech). The column was washed with 20 mM imidazole and the recombinant protein was eluted using 400 mM imidazole. The identity of the protein was verified as previously described (Kazakov et al., 2007) by matrix-assisted laser desorption ionization–time-of-flight (MALDI-TOF) mass spectrometric analysis of tryptic digests of the products by the Protein nucleic acid chemistry laboratory at the University of Leicester.

### Electrophoretic mobility shift assay (EMSA)

Fluorescently labeled DNA probe representing the putative promoter region of *tpxD* was generated using Spd1464E1FAM and Spd1464E2 primers (Table S1), and *S. pneumoniae* D39 genomic DNA. After amplification, the probe DNA was gel purified. The binding reaction was set up by mixing a constant amount of DNA promoter probe (10 nM), and increasing amounts of purified and dialyzed His-tagged recombinant CodY (0–1 μM) in 5X binding buffer (20 mM Tris-HCl pH 7.5, 30 mM KCl, 1 mM dithiothreitol (DTT), 1 mM ethylenediaminetetraacetic acid (EDTA) pH 8.0 and 10% glycerol). When required, 2 mM H_2_O_2_ and BCAA (isoleucine 1.62 mM, leucine 3.48 mM, and valine 2.77 mM) were also added into the binding buffer 20 min after addition of the DNA probe. The binding reaction was incubated at room temperature for 30 min in a total volume of 20 μl. Then, the reaction mixture was subjected to non-denaturing PAGE (8%) for 40 min at 200 V. DNA-protein complexes were visualized using a Typhoon Trio+ scanner (GE Healthcare Life Sciences) with a 526 nm short-pass wavelength filter. The image was analyzed using LI-COR image analysis software.

### Determination of CodY cysteines redox state by mass spectrometry

D39 were grown under anaerobic conditions to OD_620_ = 0.25 and then challenged with 1 mM H_2_O_2_ for 40 min while maintaining anaerobic conditions. Alkylation was performed as previously described (Hajaj et al., 2012). Briefly, *in vivo* reduced protein-thiols were blocked by resuspending phosphate buffered saline washed bacteria in urea buffer (0.1 M Tris pH 8.2, 1 mM EDTA, 8 M urea) containing 32.5 mM iodoacetic acid (IAA). Samples were then treated with DTT (3.5 mM) and incubated with iodoacetamide (IAM) (10 mM). Following alkylation, samples were resolved on 18% (w/v) SDS-PAGE, stained with Coomassie blue, and a band corresponding to ~29.5 kDa cut from the gel, reduced with 3 mM DTT (60°C for 30 min), and modified with 10 mM IAM in 100 mM ammonium bicarbonate (in the dark, room temperature for 30 min). The modified protein was then digested with modified trypsin (Promega) at a 1:10 enzyme-to-substrate ratio, overnight at 37°C. The resulting peptides were resolved by reverse-phase chromatography on 0.075 × 200-mm fused silica capillaries (J&W) packed with Reprosil reversed phase material (Dr Maisch GmbH, Germany). Mass spectrometry was performed by a Q-Exactive plus mass spectrometer (Thermo) in a positive mode using repetitively full MS scan followed by High energy Collision Dissociation (HCD) of the 10 most dominant ion selected from the first MS scan. The mass spectrometry data was analyzed using Proteome Discoverer 1.4 software Using Sequest (Thermo) algorithm searching against the *Streptococcus pneumoniae* D39 proteome from Uniprot and specific database. Semi quantitation was done by calculating the peak area of each peptide based its extracted ion currents (XICs). The area of the protein is the average of the three most intense peptides from each protein.

Results were filtered with 1% false discovery rate.

### Statistical analysis

The significance of differences was determined by the unpaired *t*-test. *p* < 0.05 was considered significant.

### Safety statement

For research involving biohazards, correct standard procedures have been carried out.

## Results

### TpxD is involved in the pneumococcal global response to H_2_O_2_

In a previous study (Hajaj et al., 2012), we showed that TpxD is an efficient H_2_O_2_ scavenger, and that its expression is up-regulated under aerobic compared to anaerobic growth conditions. In order to isolate the effect of H_2_O_2_ from other ROS formed under aerobic environment, D39 and Δ*tpxD* were challenged with sub-lethal H_2_O_2_ concentrations under anaerobic conditions. Similar viability, confirmed by colony forming unit counts, was measured in WT and the mutant cells challenged with 1 mM H_2_O_2_. Microarray profiling of D39 challenged with 1 mM H_2_O_2_ revealed that 217 genes were differentially expressed by a factor of 1.8 or more compared to unchallenged bacteria: 146 were down-regulated and 71 were up-regulated (Table S2). When the Δ*tpxD* mutant was challenged with 1 mM H_2_O_2_, global transcriptional response was not observed (Table S3). Classification of the genes affected by H_2_O_2_ in the WT into functional categories revealed that the most prominent differentially expressed genes included those associated with amino acid transport and metabolism (Table S4).

**Figure 1:**
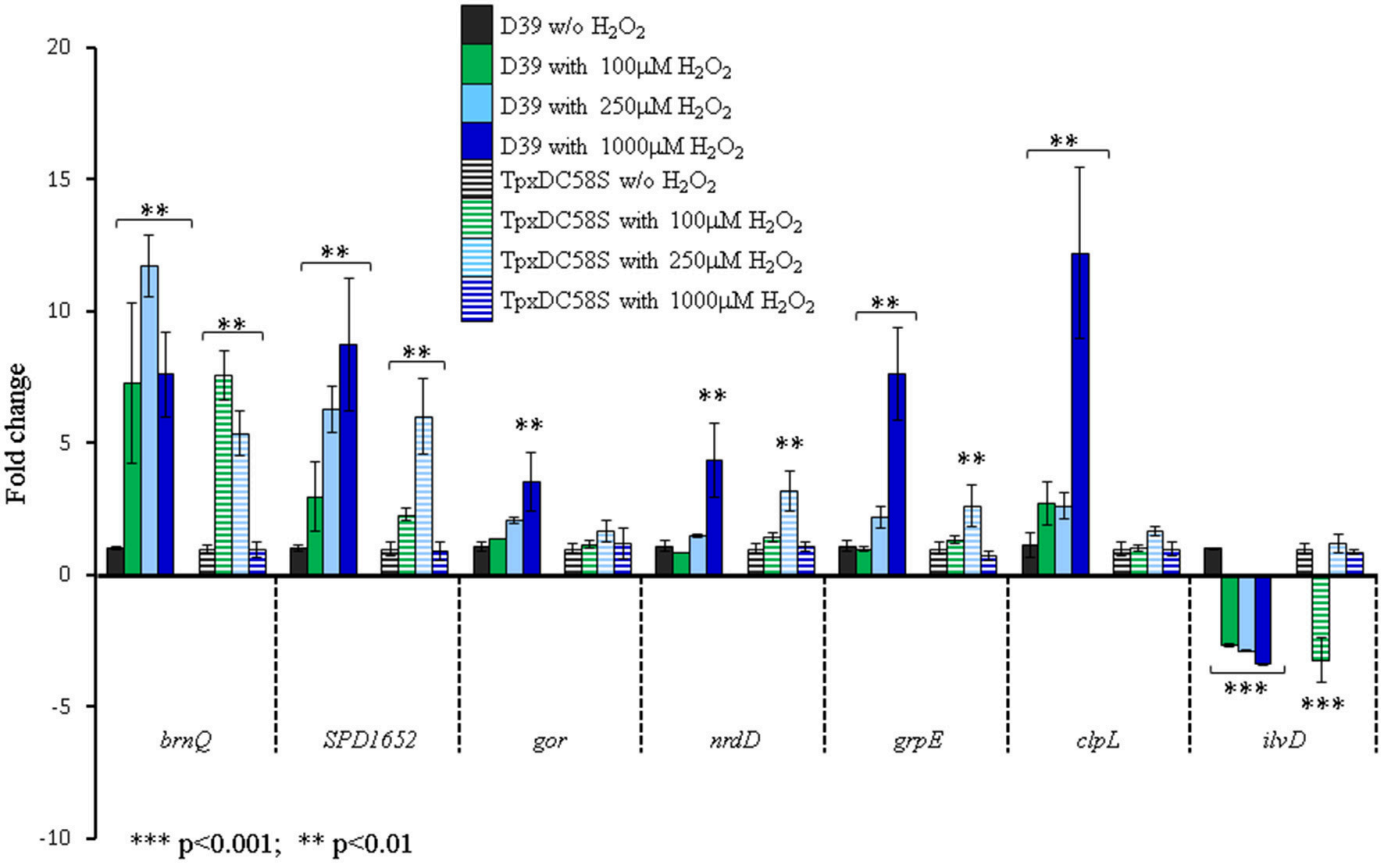
**TpxD scavenging activity is crucial for pneumococcal global response to high H_2_O_2_ levels**. Seven genes identified in the microarray to be affected by 1 mM H_2_O_2_ were selected for further examination by relative RT-PCR. D39 and TpxDC58S mutant were grown anaerobically to OD_620_ = 0.25 and then challenged with 100, 250, or 1000 μM H_2_O_2_ for 40 min. Control cultures were grown without (w/o) the addition of H_2_O_2_. Values are the mean of duplicate determinations of at least two independent experiments, and were normalized to the expression level of the relevant unchallenged strain (D39 or TpxDC58S). Bars indicate standard deviation. Significant alterations in gene expression compared to bacteria grown without H_2_O_2_ were determined using Student's *t*-test.

It was shown in eukaryotes that thiol peroxidases serve as transcriptional regulators of global gene expression in response to H_2_O_2_, in addition to enzymatic removal of H_2_O_2_ (Crooks et al., 2004). The finding that Δ*tpxD* response to H_2_O_2_ was significantly lower than that of the WT strain could originate from bacterial inability to respond to 1 mM H_2_O_2_ in the absence of TpxD scavenging activity. Alternatively, the low response could be due to TpxD role as a global transcriptional regulator in *S. pneumoniae*. Hence, we constructed a mutant in which the catalytic activity was disrupted by replacing the peroxidatic cys^58^ by serine (TpxDC58S). We selected 7 genes, whose expression was affected by 1 mM H_2_O_2_ in the WT but not in Δ*tpxD* mutant and checked their transcription levels in TpxDC58S. As shown in Figure 1, the expression levels of these genes were unaffected by 1 mM H_2_O_2_ in TpxDC58S, unlike the WT strain. However, when lower H_2_O_2_ concentrations (100 and 250 μM) were applied, TpxDC58S mutant could respond in a way similar to that of the WT strain (Figure 1). These data suggest that TpxD scavenging activity is crucial for pneumococcal global response to increased H_2_O_2_ levels. The fact that a transcriptional response to 100 and 250 μM H_2_O_2_ was induced in cells harboring inactive TpxD (TpxDC58S), rules out the possibility that TpxD serves as a transcriptional regulator.

### Identification of TpxD promoter elements

**Figure 2:**
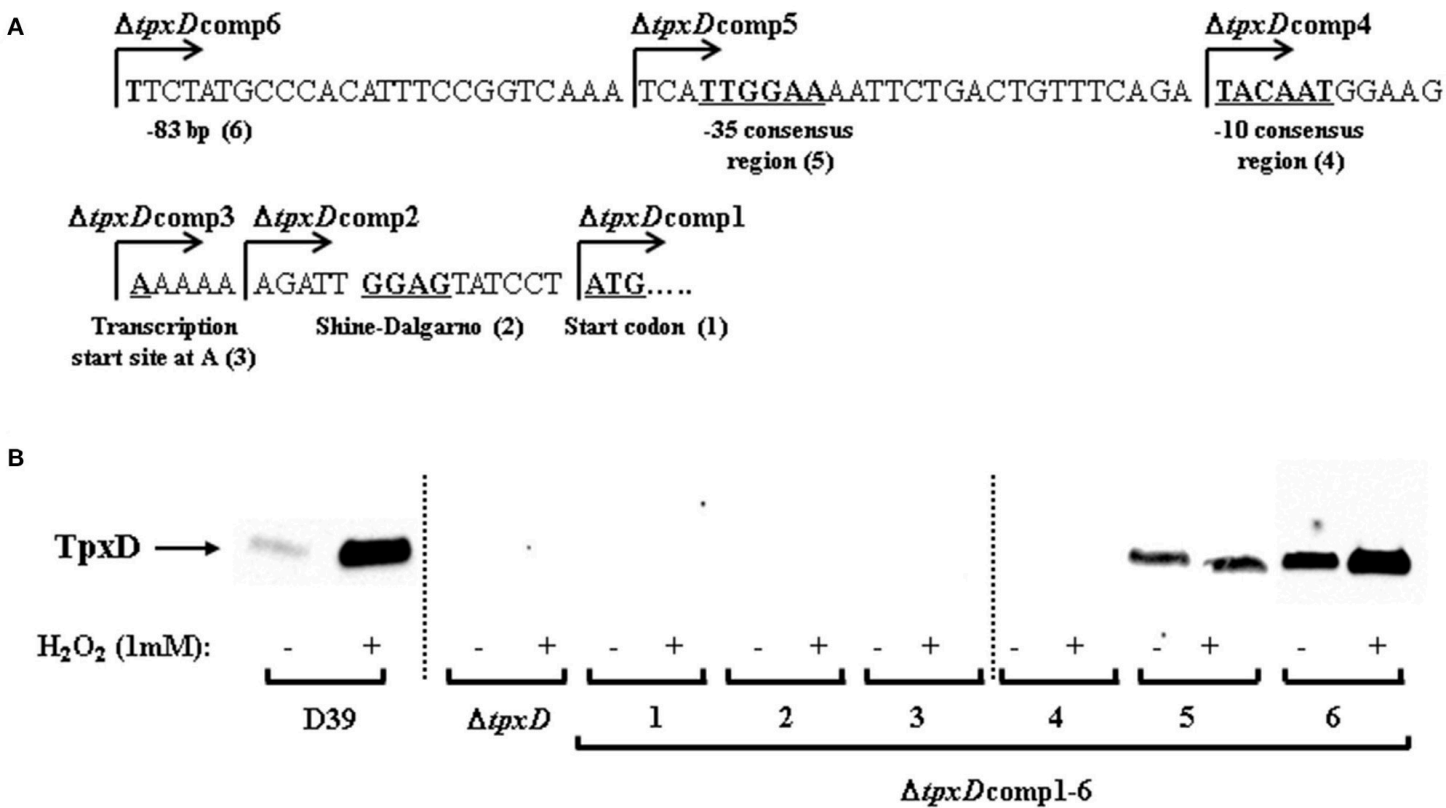
**Identification of *tpxD* regulatory promoter elements. (A)** *In silico* analysis of *tpxD* proximal promoter region. Identification of *tpxD* promoter elements using BPROM algorithm: ATG start codon (1); Shine Dalgarno (2); transcription start site at A (3); −10 and −35 elements of the σ^70^ promoter (4 and 5, respectively). The first nucleotide included in each of the Δ*tpxD*comp mutants (1–6) is indicated by arrow. **(B)** Influence of *tpxD* putative regulatory elements on TpxD expression levels under H_2_O_2_ stress. TpxD levels, determined by Western blotting, were measured in D39, Δ*tpxD* and Δ*tpxD*comp1-6 (described in Figure 2A). Bacteria were grown anaerobically to OD_620_ = 0.25 and then challenged with 1 mM H_2_O_2_ for 40 min. Separated proteins were electroblotted onto 0.45 μm nitrocellulose membrane, and stained with Ponceau S to ensure equal loading, before incubation with antibodies. Membranes were exposed for long time intervals to ensure the absence of bands in Δ*tpxD*comp1-4 and are lined up, as indicated in the figure. Densitometry was performed using ImageJ software.

We have shown that TpxD expression and synthesis were significantly increased following a challenge with H_2_O_2_ (Hajaj et al., 2012). To discover the specific DNA-elements responsible for TpxD synthesis and regulation by H_2_O_2_, *in silico* tools were applied. BPROM algorithm predicted 5 core elements upstream of *tpxD*-coding sequence (Figure 2A). Based on these predictions, we constructed 6 genetically modified strains, in which Δ*tpxD* was *cis*-complemented with an intact copy of *tpxD-*coding sequence, preceded by a variable length of *tpxD* proximal upstream region: Δ*tpxD*comp1 harboring only *tpxD-*coding sequence; Δ*tpxD*comp2 harboring the sequence starting from the predicted Shine-Dalgarno element and up to and including *tpxD-*coding sequence; Δ*tpxD*comp3 harboring the sequence starting from the predicted transcription start site and up to and including *tpxD-*coding sequence; Δ*tpxD*comp4 and 5 harboring the sequence starting from the predicted −10 and −35 consensus regions of the promoter, respectively, and up to and including *tpxD-*coding sequence; Δ*tpxD*comp6 harboring *tpxD-*coding sequence plus a region of 83 bp upstream of the gene (Figure 2A). Lysates of Δ*tpxD*comp1-6 strains, grown anaerobically, were subjected to Western blotting using polyclonal antibodies against TpxD. No TpxD-band was seen in Δ*tpxD*comp1-4 strains (Figure 2B). In Δ*tpxD*comp5, basal TpxD protein level was detected, indicating that the Shine-Dalgarno, transcription start site, −10 and −35 consensus regions are essential for TpxD synthesis. However, Δ*tpxD*comp5 did not show any up-regulation in response to 1 mM H_2_O_2_compared to the WT strain (Figure 2B). Only Δ*tpxD*comp6 showed a significant increase in TpxD synthesis following a challenge with H_2_O_2_as measured by densitometry: 1.70 ± 0.13-fold, *p* < 0.05 (Figure 2B). These results point out that the regulatory elements essential for *tpxD* up-regulation by H_2_O_2_ are located within the immediate 83 bp upstream of *tpxD-*coding sequence.

### CodY binding motif in the proximal upstream region of *tpxD*

The next step was to identify a candidate TF responsible for *tpxD* up-regulation under H_2_O_2_ stress. To this end, we clustered the genes, shown in the microarray to be affected by H_2_O_2_, according to their specific regulator, by using RegTransBase database (Fomenko et al., 2011). Clustering was done solely to highlight a potential TF, being aware of the fact that H_2_O_2_ invokes a general stress response, involving many transcription factors and genes, making it very hard to distinguish direct from indirect effects of H_2_O_2_. This analysis yielded 14 TF-clusters covering 60/217 of the genes found by the microarray to be significantly affected by 1 mM H_2_O_2_ (Table 1). It should be noted that 16 of the 60 genes were classified in 2 TF-clusters (based on RegTransBase database) and that the regulon grouping of some genes is controversial.

**Table 1:** **Clustering of genes significantly affected by 1 mM H_2_O_2_, by at least a factor of 1.8, according to their specific TF**.

**Transcription factor**	**D39 locus tag^a^**	**No of genes**
Transcriptional repressor, CodY	SPD_1412	21
Catabolite control protein A, CcpA	SPD_1797	12
DNA-binding response regulator, YycF	SPD_1085	10
Transcriptional regulator, MarR family protein, FabT	SPD_0379	6
DNA-binding response regulator	SPD_0344	5
DNA-binding response regulator, CiaR	SPD_0701	5
Transcriptional regulator, MerR family protein	SPD_0447	3
Iron-dependent transcriptional regulator, PsaR	SPD_1450	3
Heat-inducible transcription repressor, HrcA	SPD_0458	3
Transcriptional regulator, CtsR	SPD_2023	2
*adc* operon repressor, AdcR	SPD_2000	2
Transcriptional regulator, NrdR	SPD_1523	2
Arginine repressor, ArgR	SPD_1904	1
Maltose operon transcriptional repressor, MalR	SPD_1938	1

a *Gene number refers to D39 locus tags*.

**Figure 3:**
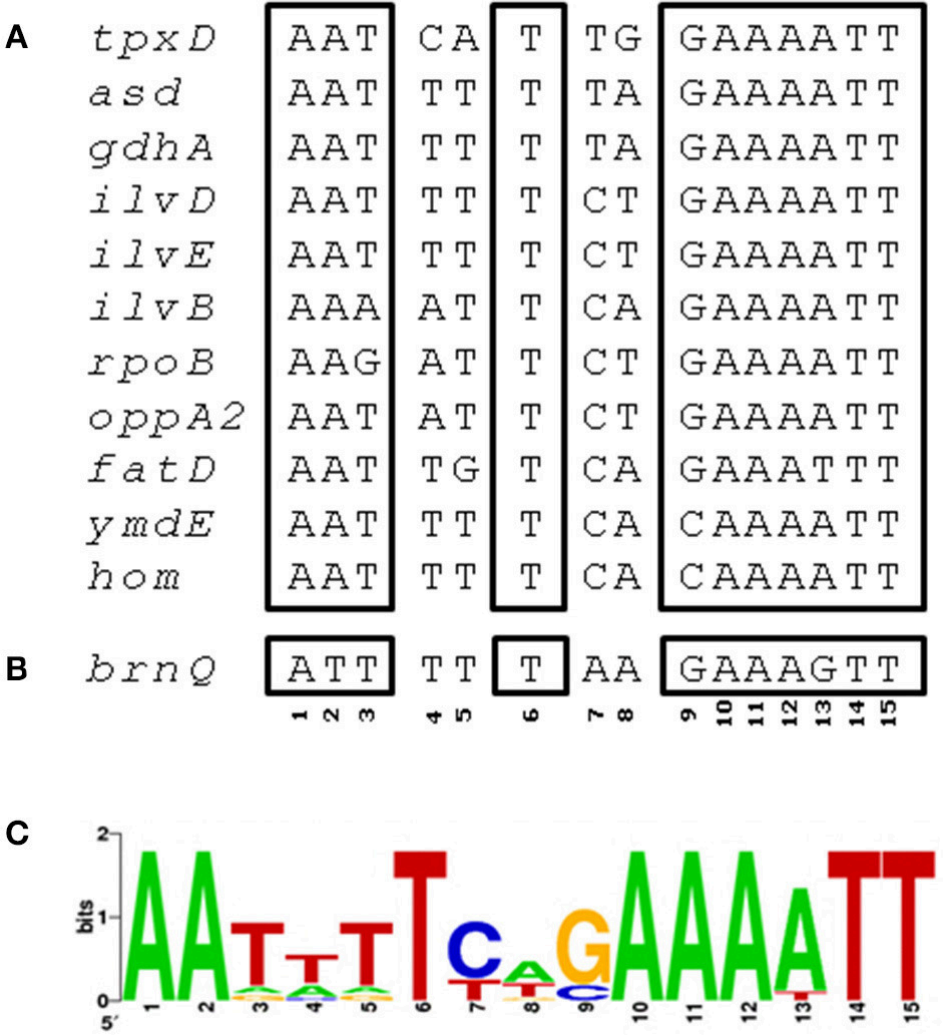
**CodY binding site in *tpxD* promoter region. (A)** ClustalW was used to align the 83 bp upstream to *tpxD*-coding sequence with several pneumococcal genes known to be regulated by CodY. A region of 15 bp, between −26 and −40, upstream to *tpxD*-transcription start site, with high homology to CodY binding motif in *L. lactis*, is presented. **(B)** Putative CodY binding site in the upstream region of *brnQ*. **(C)** Position specific weight matrix for *S. pneumoniae* CodY binding motif deduced from CodY-regulated, established and putative genes (Figure 3A), identified by bioinformatic analysis of the microarray data, using RegPrecise database. The matrix (created at http://weblogo.berkeley.edu/) shows the relative frequency of occurrence of each of the 4 nucleotides (A, T, C, G) at each of the 15 positions of the motif.

We then used ClustalW to check whether *tpxD* upstream sequence contains a putative binding site for one of the TFs reported in Table 1. This analysis identified a CodY-binding site of 15 bp, between −26 and −40, upstream of *tpxD-*transcription start site (AATCATTGGAAAATT) which is highly homologous with CodY-binding motifs of pneumococcal genes known to be under CodY regulation (Figure 3). This putative binding site shares 73% homology with CodY-consensus sequence in *Lactococcus lactis* (den Hengst et al., 2005; Guedon et al., 2005).

**Figure 4:**
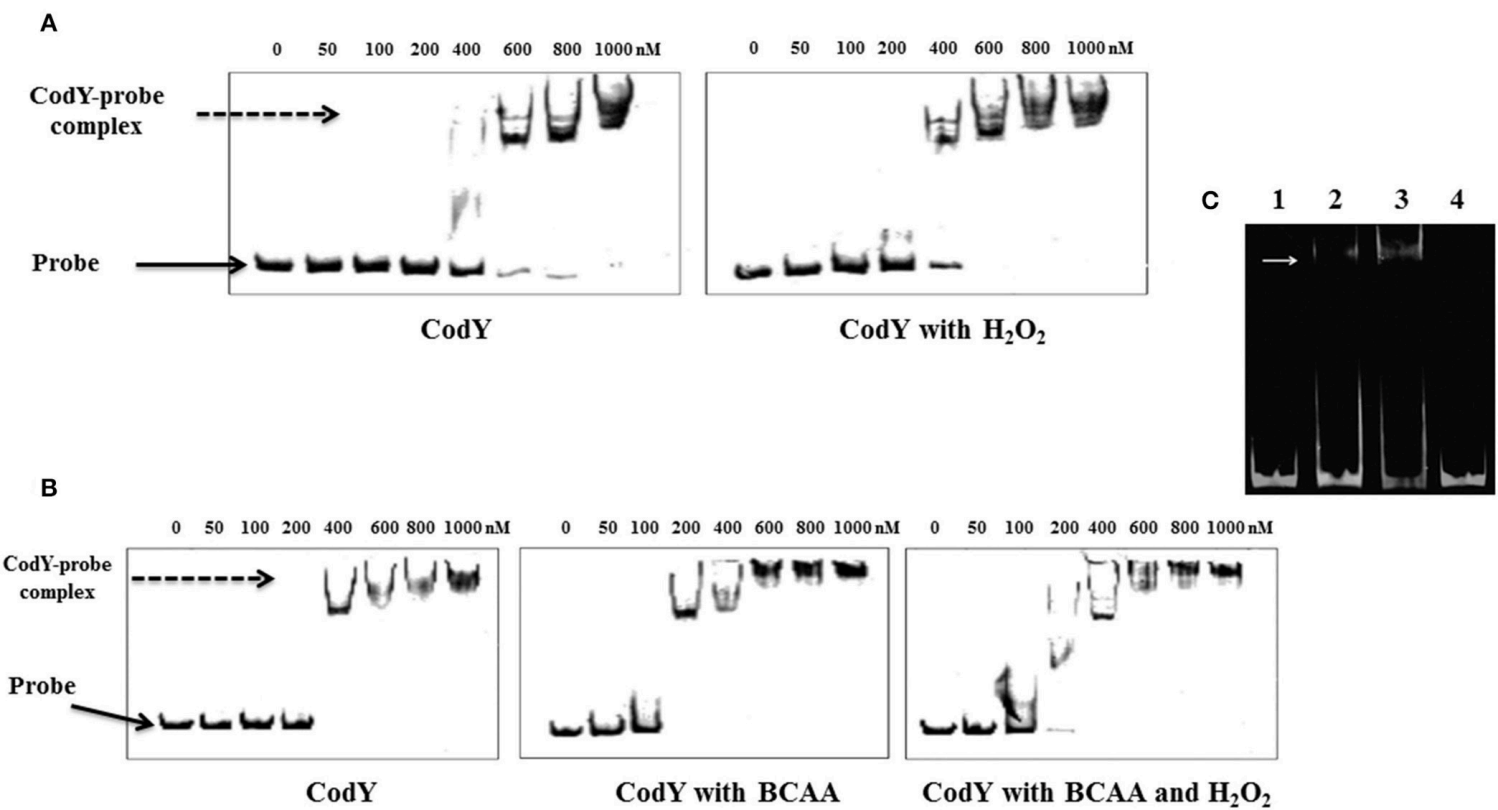
**CodY interaction with the regulatory region of tpxD**. EMSA assay was used to confirm the presence of CodY binding site in tpxD regulatory region: Effect of H_2_O_2_ **(A)** and BCAA **(B)**. 10 nM of fluorescently labeled DNA probe was mixed with increasing concentration of recombinant CodY, and incubated for 30 min at room temperature. When required, 2 mM H_2_O_2_ and/or BCAA (isoleucine 1.62 mM, leucine 3.48 mM, and valine 2.77 mM) were added into the binding buffer. DNA-protein complexes were separated by electrophoresis on non-denaturing PAGE (8%). In all gels, Lane 1 contains DNA probe alone, indicated with a black arrow. DNA-protein interaction is seen as an upward shift of DNA probe, indicated with a dotted arrow. **(C)** Lane 1, 50 ng approximately of the 81 bp DNA fragment alone, lane 2 and 3, DNA plus 0.6- and 1-mM recombinant CodY, respectively. Lane 4, DNA probe, excluding 15 bp putative CodY binding site plus 1 mM recombinant CodY. White arrow indicates the position of DNA-protein complex. EMSA was performed using Molecular Probes fluorescence-based EMSA kit (Invitrogen). Briefly, 5X FY binding buffer (20 mM Tris-HCl pH 7.5, 30 mM KCl, 1 mM DTT, 1 mM EDTA pH 8.0 and 10% v/v glycerol) was prepared to incubate the promoter probe and protein. The binding reaction was set up by mixing a constant amount of target promoter probe (~30 ng), and increasing amounts (0.1–0.5 μM) of purified and dialyzed His-tagged protein. The binding reaction was incubated at room temperature for 20 min in a total volume of 20 μl, and then analyzed on an 8% w/v non-denaturing polyacrylamide gel. After electrophoresis, gels were stained with SYBR® Green EMSA gel stain (Invitrogen) and visualized using a Typhoon Trio+ scanner (GE Healthcare Life Sciences) with a 526 nm short-pass wavelength filter.

We then expressed and purified the pneumococcal CodY protein with the intent to test its *in vitro* binding to the motif upstream of *tpxD*-coding sequence. EMSA results showed indeed that CodY interacts with the DNA probe containing this region in a concentration dependent manner (Figure 4). CodY binding to *tpxD* upstream region was specific, first, as we demonstrated previously that CodY does not bind to the *psaR* promoter, which is known not to be regulated by CodY (Hendriksen et al., 2008). Second, we deleted the putative CodY binding sequence in the upstream region of *tpxD*, and found that the binding was abolished (Figure 4C). The effect of H_2_O_2_ on CodY binding to the motif upstream of *tpxD*-coding sequence was checked by the incubation of CodY with 2 mM H_2_O_2_ prior to the addition of target DNA. This experiment resulted in increased CodY affinity to its target: 400 nM were sufficient for CodY binding to DNA in the presence of H_2_O_2_ whereas 600 nM were needed without H_2_O_2_ (Figure 4A). CodY-DNA binding activity was shown to be increased by BCAA (Brinsmade et al., 2010). When the reaction mixture was supplemented with BCAA, DNA binding was observed already at 200 nM. In this case addition of H_2_O_2_ had no impact (Figure 4B). These data indicate that the 15 bp sequence serves as CodY binding site. Furthermore, H_2_O_2_ increases CodY binding to its DNA target under conditions where BCAA are not present.

### Effect of CodY-mutation on *tpxD* expression

Our attempts to inactivate *codY* were unsuccessful, probably due to its essentiality. Hence we used a *codY*-deletion mutant harboring suppressing mutations inactivating the *fatC* and *amiC* genes (Caymaris et al., 2010). To elucidate the influence of CodY on TpxD expression, bacteria were grown in CDM without BCAA. Under anaerobic conditions, *tpxD* expression was significantly reduced in Δ*codY* compared to its wild type, 2.43 ± 0.25-fold reduction, indicating that CodY is an activator of *tpxD*, even without BCAA. A 4.00 ± 0.12-fold reduction in *tpxD* expression was measured when Δ*codY* cells were challenged with 1 mM H_2_O_2_. Hence we conclude that CodY is required for *tpxD* up-regulation under high H_2_O_2_ levels.

### Effect of BCAA on *tpxD* expression

**Figure 5:**
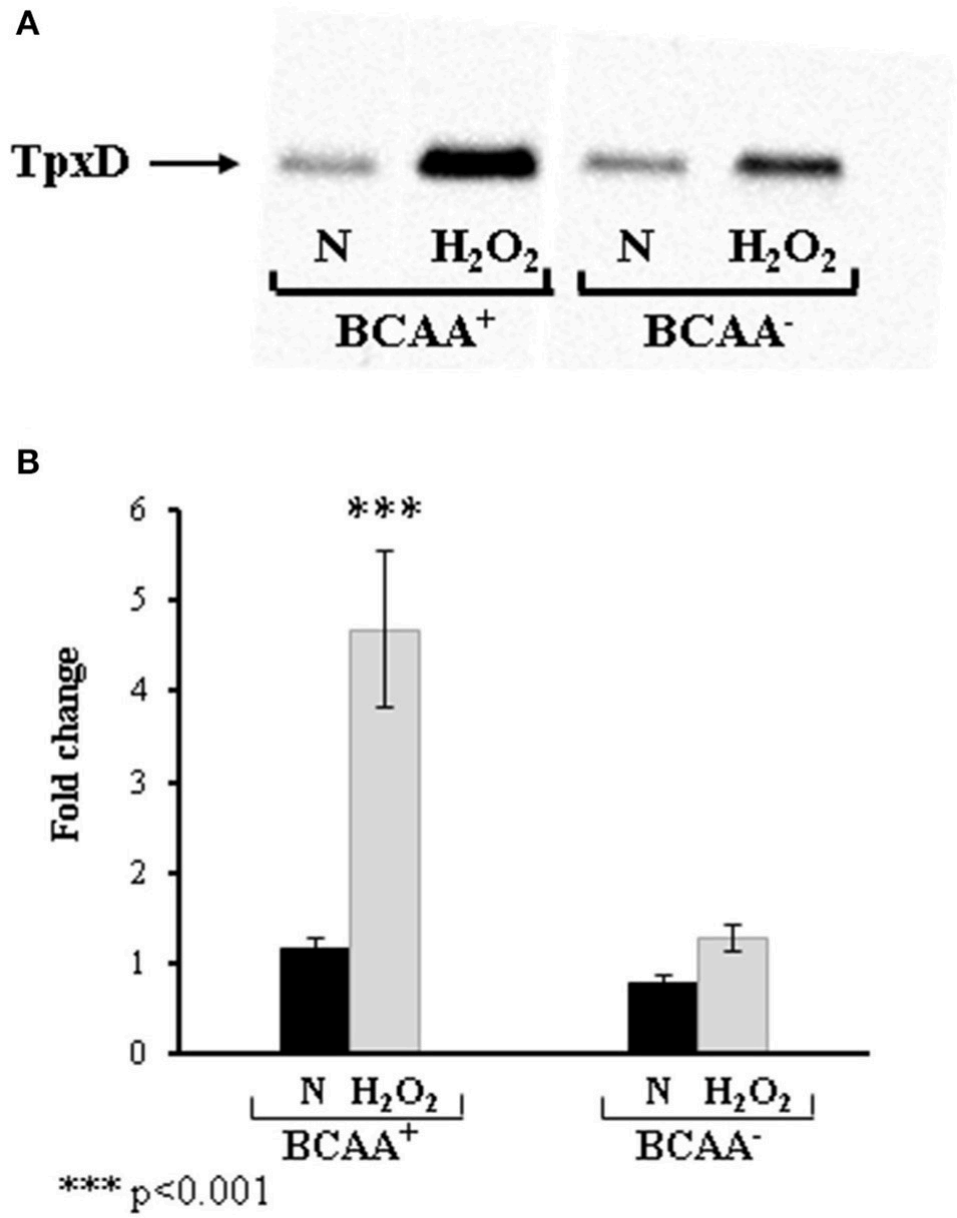
**Effect of BCAA on TpxD expression in bacteria challenged with H_2_O_2_**. TpxD synthesis and expression levels were determined by Western blotting **(A)** and by RT-PCR **(B)** in D39 grown anaerobically (N) in CDM with (BCAA+) or without (BCAA-) BCAA. Bacteria were grown to OD_620_ = 0.25 and then challenged with 1 mM H_2_O_2_ for 40 min. Control cultures were incubated without the addition of H_2_O_2_. Separated proteins were electroblotted onto 0.45 μm nitrocellulose membrane, and stained with Ponceau S to ensure equal loading, before incubation with antibodies. Values are the mean of duplicate determinations of at least two independent experiments, and were normalized to the expression level of D39 grown anaerobically in medium containing BCAA. Bars indicate standard deviation. Significant alterations in gene expression compared to bacteria grown without H_2_O_2_ were determined using Student's *t*-test.

To elucidate the role of BCAA in CodY regulation of TpxD expression and synthesis, D39 were grown in the absence of BCAA (Figure 5). This experiment revealed that BCAA had no effect on the basal level of TpxD under anaerobic growth conditions. In addition, TpxD up-regulation by H_2_O_2_ was less pronounced in bacteria grown without BCAA. These data indicate that BCAA are required for CodY-mediated, TpxD up-regulation by H_2_O_2_. We then checked whether BCAA itself affect *tpxD* expression in a mechanism independent of CodY. To this end, *tpxD* expression was assessed in Δ*codY* grown with or without BCAA and challenged with H_2_O_2_. This comparison revealed that the presence of BCAA was accompanied by a 2.27 ± 0.20-fold reduction in *tpxD* expression in Δ*codY*, suggesting that in the absence of CodY, BCAA interfere with *tpxD* expression in as yet unresolved mechanism.

### The BCAA transporter *brnQ* is a putative CodY regulated gene

BCAA intracellular level is determined by their biosynthesis through the *ilv* operon (Brinsmade et al., 2010) and by their transport through specific transporters (Belitsky, 2015). The microarray data showed that the BCAA- biosynthetic enzymatic machinery (*ilv*) was down-regulated following a challenge with H_2_O_2_ (Table S2). Concomitantly, the expression of *brnQ*, a BCAA transport system II carrier protein, showed a remarkable increase, 7.6-fold, following a challenge with 1 mM H_2_O_2_ (Figure 1), probably to compensate for the decrease in BCAA biosynthesis. Noteworthy is the fact that a Δ*brnQ* mutant could not overcome a challenge with 0.5–1.0 mM H_2_O_2_ (data not shown), indicating the importance of BCAA for oxidative stress resistance. ClustalW analysis revealed a putative CodY binding site in the upstream region of *brnQ* (Figure 3). Taken together the fact that CodY functions as a transcriptional repressor of the *ilv* operon (Hendriksen et al., 2008) on the one hand, and our the finding of a putative CodY binding site upstream of *brnQ* coding sequence on the other, implies that CodY is involved in maintaining homeostatic levels of BCAA in the cell.

### H_2_O_2_ specifically oxidizes CodY cysteines

Microarray data showed that *codY* expression was increased by 1.8-fold in the presence of 1 mM H_2_O_2_ (Table S2). Validation by RT-PCR and Western blotting showed no significant change in CodY expression (Figure S1). This led us to conclude that CodY regulation by H_2_O_2_ is carried out by a different mechanism. Hence we checked the effect of H_2_O_2_ on the redox state of CodY-cysteines by a thiol double trapping method followed by mass spectrometry. Our data show that under anaerobic conditions, the 2 CodY-cysteines were always in the reduced state, as the pick area of the oxidized, IAM-alkylated cysteines, was zero. Following a challenge with H_2_O_2_, the pick area of the reduced, IAA-labeled cysteines, was 13E7 whereas that of the oxidized, IAM labeled cysteines, was 3.75E7. These data indicate that H_2_O_2_ triggers the oxidation of the 2 cysteines in about 1/4 of CodY molecules, which might influence CodY activity.

## Discussion

Hydrogen peroxide is well known as an oxidant that can react with various cellular targets. At low levels, H_2_O_2_ functions as a signaling agent, whereas at high levels, H_2_O_2_ inhibits the growth of bacteria and induces cell death (Imlay and Linn, 1986; Johnston et al., 2015). Homeostatic control of this oxidant is maintained by several oxidant-scavenging enzymes. We have previously shown that *tpxD* encodes a functional thiol peroxidase, involved in H_2_O_2_ homeostasis in *S. pneumoniae* (Hajaj et al., 2012). In the current study we show that pneumococcal response to high H_2_O_2_ levels depends on TpxD, and that *tpxD* expression is regulated by the global transcription factor CodY.

Microarray analysis revealed a global transcriptional response to high H_2_O_2_ concentration in the WT but not in Δ*tpxD*-null cells. To verify whether this global response depends on TpxD activity, the peroxidatic cysteine^58^ was replaced by serine, TpxDC58S. Similar substitution of the peroxidatic Tpx-cysteine in *E. coli* resulted in an inactive enzyme (Baker and Poole, 2003), and in a peroxide-sensitive phenotype in *Enterococcus faecalis* (La Carbona et al., 2007). Based on the sequence similarity between *S. pneumoniae* TpxD and *E. coli* Tpx (56.9%) as well as between *S. pneumoniae* TpxD and *E. faecalis* Tpx (55.1%), and their established H_2_O_2_ scavenging activity, we surmise that the catalytic activity of TpxDC58S is ablated. The expression of 7 genes, shown to be affected by 1 mM H_2_O_2_ in the WT but not in Δ*tpxD* mutant, was checked in TpxDC58S. These genes showed no response to 1 mM H_2_O_2_, but were differentially expressed upon exposure to 100 and/or 250 μM H_2_O_2_. These findings indicate that TpxD scavenging activity is required for the bacterial response to increased H_2_O_2_ concentrations. Of note is the fact that H_2_O_2_ can be reduced to OH· by Fe^+2^ via the Fenton reaction under both aerobic and anaerobic conditions (Henle et al., 1996). Hence, some of the changes in gene expression could originate from the effect of OH· rather than H_2_O_2_. The effect of the Fenton reaction on global gene expression needs further investigation.

In a previous study (Hajaj et al., 2012) we found that TpxD expression is modulated in accordance with H_2_O_2_ levels: in a WT strain recovered from the nasopharynx of mice following intranasal infection, *tpxD* expression level was higher than that measured in bacteria recovered from the less aerated niche of the blood, in agreement with the *in vitro* expression levels in cells grown under aerobic vs. anaerobic conditions or challenged with H_2_O_2_. A recent publication (Lisher et al., 2017) demonstrated that *tpxD* level is increased by 6.8 following growth with limited aeration compared with growth under anaerobic condition. These authors show that TpxD plays a major role in preventing endogenous protein sulfenylation by H_2_O_2_, reinforcing our conclusion that TpxD is required for the bacterial response to H_2_O_2_.

Bioinformatic tools pointed toward CodY as a potential TF mediating the cellular response to H_2_O_2_, since expression of 21 genes known to be under its regulation were repressed/activated (Table S5). CodY is a highly conserved global regulator in many low G+C Gram positive bacteria. Targets of CodY include operons involved in amino acid metabolism, particularly BCAA, as well as in proteolysis and peptide uptake and degradation (Geiger and Wolz, 2014). CodY is also a repressor of the iron transport operon *fatD*–*fatC*–*fecE*–*fatB* and the Ami-Obl oligopeptide transporter (Hendriksen et al., 2008), and an activator of the essential iron storage-peroxide resistance Dpr protein (Pericone et al., 2003). Our attempts to inactivate *codY* without disrupting additional genes were unsuccessful, in line with the data published by Caymaris et al. (2010): *codY* inactivation results in derepression of iron uptake by the two mentioned transporters and depletion of iron storage by Dpr, leading to a severe oxidative stress (Hendriksen et al., 2008). Consequently, simultaneous mutations in *fatC* and *amiC* arises to prevent the accumulation of high levels of iron, and thereby limit the formation of reactive oxygen intermediates through the Fenton reaction (Caymaris et al., 2010; Johnston et al., 2015). We therefore used a *codY*-deletion mutant, possessing suppressing mutations in *fatC* and *amiC*, and found that CodY is an activator of *tpxD*. A putative CodY binding site was found in the proximal region of *tpxD-*coding sequence. Genetic engineering techniques and EMSA assays confirmed the presence of an active CodY binding site. This site overlaps the −35 consensus region of *tpxD*, suggesting that CodY belongs to Class II activators, which bind to sites that overlap the target promoter −35 region, and in most cases activate transcription by making a direct interaction with domain 4 of the RNA-polymerase subunit (Dove et al., 2003). H_2_O_2_ Increased CodY binding to *tpxD* regulatory sequence, indicating that H_2_O_2_ positively regulates *tpxD*. *Streptococcus mutans tpx* was also found to be regulated in a positive manner, although not by CodY but by the oxidative stress regulator SpxA1(Kajfasz et al., 2015).

BCAA are known to enhance CodY binding to DNA (Brinsmade et al., 2010). BCAA intracellular level is determined by their biosynthesis through the *ilv* operon (Brinsmade et al., 2010) and by their transport through specific transporters (Belitsky, 2015). The biosynthetic pathway can be affected by oxidative stress; in particular, the [4Fe-4S] prosthetic group of IlvD has been shown to react with H_2_O_2_ in *E. coli* (Flint et al., 1993). Henard et al. suggested that oxidation of the redox active center of IlvD in *Salmonella* is expected to decrease the biosynthesis of BCAA (Henard et al., 2010). If this is the case in *S. pneumoniae*, it may explain the down-regulation of *ilvD* as well as additional *ilv* genes by CodY, presumably to prevent the expression of genes belonging to a pathway that will subsequently undergo oxidation, and thus will be inactive. Bioinformatic analysis identified a putative CodY binding site in the upstream region of the BCAA transporter, *brnQ*, similar to that found in *B. subtilis* (Belitsky, 2015), suggesting that CodY controls both BCAA biosynthesis and transport, to ensure a steady supply of these essential amino acids. The reciprocity between CodY and BCAA constitutes an indirect auto-regulatory loop that allows the fine-tuning of BCAA level and CodY activity in *S. pneumoniae*, as published before for *B. subtilis* (Belitsky, 2015). These authors have shown that the basal level of BCAA achieved by intracellular synthesis supports a low level of CodY activity that is sufficient for partial regulation of some genes. However, maximal CodY activity is observed only in the presence of BCAA-containing amino acid mixtures. Thus, uptake of exogenous BCAA is critical for attaining conditions favoring CodY activation, and the efficiency of such uptake is likely to determine the level of CodY activation (Belitsky, 2015). However, in *Listeria monocytogenes* CodY was shown to be active even when bacteria were starved for BCAA (Lobel and Herskovits, 2016). These authors suggest that additional factors are involved in mediating CodY binding to its DNA targets. In line with this are our EMSA results showing that H_2_O_2_ enhances CodY binding to *tpxD*- target DNA in the absence of BCAA, resulting in *tpxD* up-regulation. CodY harbors a CXXC motif, thought to facilitate disulfide bond formation when exposed to an oxidizing environment (Kajfasz et al., 2015). We show that H_2_O_2_ specifically oxidizes these 2 adjacent cysteines and speculate that CodY-cysteines oxidation triggers a conformational change that ultimately facilitates CodY interaction with its DNA binding site. The finding that only 1/4 of CodY molecules undergo oxidation at high H_2_O_2_ levels suggests that *tpxD* expression is strictly controlled, to ensure homeostatic levels of H_2_O_2_ in the cell. Further research is needed to establish this mechanism.

**Figure 6:**
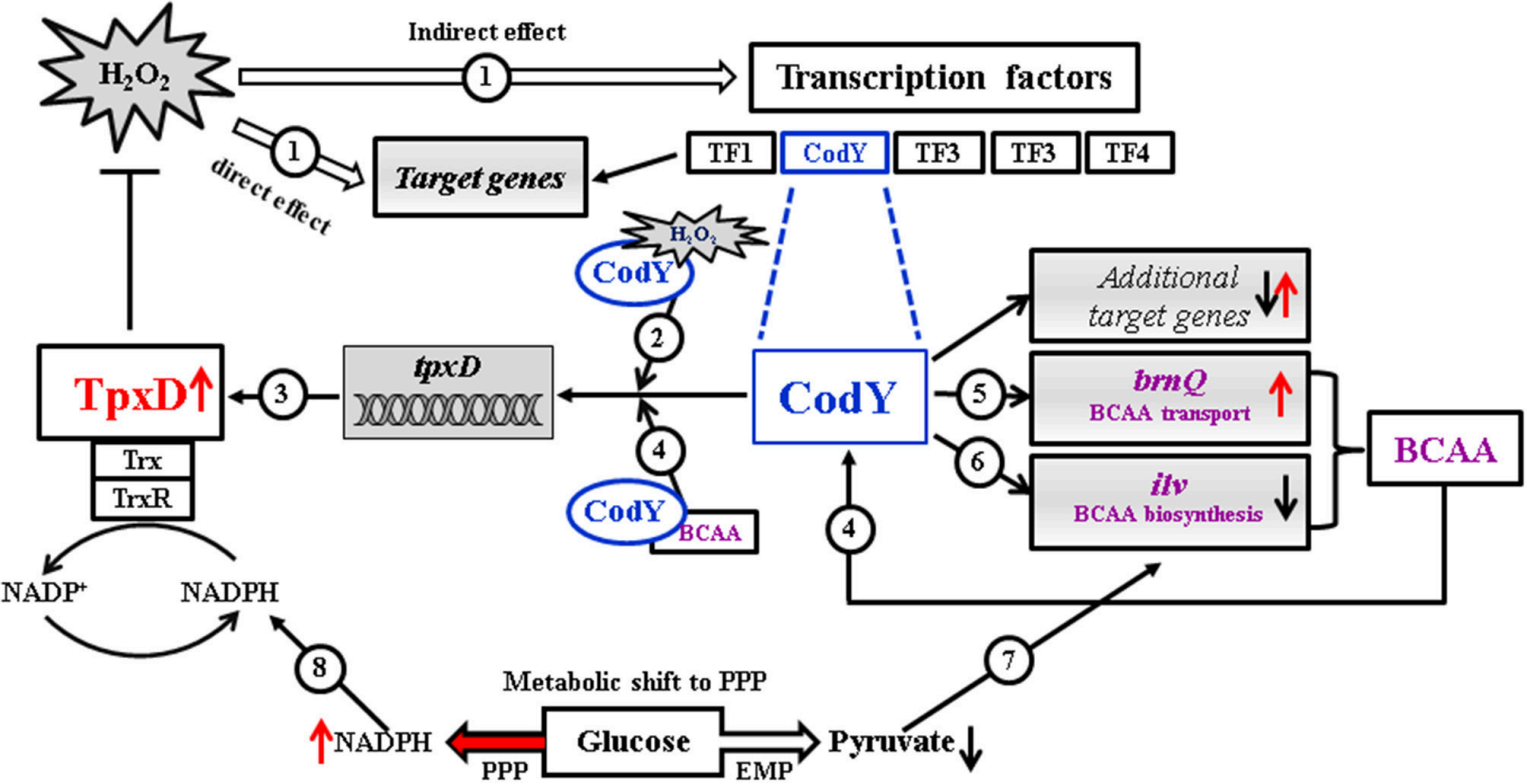
**Proposed schematic model illustrating the contribution of TpxD and CodY to the pneumococcal global transcriptional response to H_2_O_2_**. Under oxidative stress conditions H_2_O_2_ activates directly or indirectly a cascade of genes and transcription factors (TF), among them CodY (1). We speculate that CodY activation by H_2_O_2_ is due to a conformational change originating from specific cysteine oxidation (2). As a result, CodY binding to *tpxD* regulatory sequence is enhanced, leading to TpxD up-regulation (3), thereby preventing the accumulation of high H_2_O_2_ levels. BCAA are known to enhance CodY binding to DNA (4), thereby inducing *tpxD* expression (3). Under oxidative stress conditions, CodY modulates the intracellular level of BCAA through the up-regulation of the BCAA transporter, *brnQ* (5) and down-regulation of the BCAA biosynthetic machinery (*ilv*) (6). Consequently, the need for pyruvate, which is the central precursor in BCAA biosynthesis, is expected to decrease (7) leading to a shift in glucose catabolism toward the pentose phosphate pathway (PPP). This metabolic shift will result in increased NADPH formation (8), thus enhancing TpxD recycling by the thioredoxin-thioredoxin reductase system.

We present a schematic model (Figure 6) illustrating the contribution of TpxD and CodY to the pneumococcal global transcriptional response to H_2_O_2_. Under oxidative stress, H_2_O_2_ activates directly or indirectly a cascade of genes and transcription factors, among them CodY. We speculate that CodY activation by H_2_O_2_ is due to a conformational change originating from specific cysteine oxidation. As a result, CodY binding to *tpxD* regulatory sequence is enhanced, leading to TpxD up-regulation, thereby preventing the accumulation of high H_2_O_2_ levels. In addition, we show that under oxidative stress conditions, CodY modulates the intracellular level of BCAA (known to enhance CodY binding to DNA) through the up-regulation of the BCAA transporter, *brnQ*, and down-regulation of the BCAA biosynthetic machinery. Consequently, the need for pyruvate, which is the central precursor in BCAA biosynthesis, is expected to decrease. Hence, glucose catabolism will be shifted from the Embden-Meyerhof-Parnas (EMP) pathway toward the pentose phosphate pathway (PPP) resulting in NADPH formation, via the oxidative branch (Krüger et al., 2011). In line with this are the findings in *E. coli* (Sandoval et al., 2011) showing that high ROS levels lead to overexpression of glucose-6-phosphate dehydrogenase (*zwf*), the enzyme directing glucose to PPP. Increased NADPH levels are essential under high H_2_O_2_ levels, since TpxD activity relies on the recycling of its catalytic cysteine residues by the thioredoxin-thioredoxin reductase system.

Results presented in this manuscript attribute a role for CodY in maintaining homeostatic levels of H_2_O_2_ in *S. pneumoniae*. This role is achieved by the regulation of *tpxD*, to enable bacterial survival under oxidative stress conditions. We also demonstrate that some of the genes involved in oxidative stress response, which are also known to be regulated by CodY, were not affected by high H_2_O_2_ levels in *tpxD* mutants Δ*tpxD* and TpxDC58S), signifying that active-TpxD is required for CodY function as a TF under oxidative stress conditions.

## Author contributions

Substantial contributions to the conception or design of the work; or the acquisition, analysis, or interpretation of data for the work: BH, HY, SS, XZ, RB, ST, OK, NP. Drafting the work or revising it critically for important intellectual content: BH, HY, SS, XZ, RB, ST, OK, NP. Final approval of the version to be published: BH, HY, SS, XZ, RB, ST, OK, NP. Agreement to be accountable for all aspects of the work in ensuring that questions related to the accuracy or integrity of any part of the work are appropriately investigated and resolved: BH, HY, SS, XZ, RB, ST, OK, NP.

### Conflict of interest statement

The authors declare that the research was conducted in the absence of any commercial or financial relationships that could be construed as a potential conflict of interest.
