# Supplementary material for: CodY Regulates Thiol Peroxidase Expression as Part of the Pneumococcal Defense Mechanism against H2O2 Stress
Source: Front Cell Infect Microbiol. 2017 May 24;7:210. doi: 10.3389/fcimb.2017.00210 (PMC5443158; doi:10.3389/fcimb.2017.00210)
Supplement: Supplementary file 1 [file Table1.DOCX]

**Table S1. Oligonucleotide primers used in this study**

| **Primer ID^a^** | **Primer Sequence (5’-3’)** | **Purpose of use** |
| --- | --- | --- |
| ***Mutation and genetic complementation*** | | |
| SPD0546RR | ATGCCCAAGCCTACTATTTACAG | Δ*brnQ* |
| SPD0546LF | TATTCACGAACGAAAATCGATCCACGATTAGGAGTCTG |  |
| SPD0546RF | AACAATAAACCCTTGCATAACAATAATCACTAATACTCTTCG |  |
| SPD0546LR | GTTGTTACGGGCAATTCAATCTTCTC |  |
| Tpxcomp1 | cgccatggATGGTAACTTTTCTCGGAAATCC | Δ*tpxD*comp1 |
| Tpxcomp2 | cgccatggAGATTGGAGTATCCTATGG | Δ*tpxD*comp2 |
| Tpxcomp3 | cgccatggAAAAAAGATTGGAGTATCC | Δ*tpxD*comp3 |
| Tpxcomp4 | cgccatggTACAATGGAAGAAAAAAG | Δ*tpxD*comp4 |
| Tpxcomp5 | cgccatggTCATTGGAAAATTCTGAC | Δ*tpxD*comp5 |
| Tpxcomp6 | cgccatggTTCTATGCCCACATTTCC | Δ*tpxD*comp6 |
| TPX-S1 | CAGGCATCAGCTCAACTCAAACAC | TpxDcys58ser |
| TPX-S2 | GAGTTGAGCTGATGCCTGTATCG |  |
| malF | GCTTGAAAAGGAGTATACTT | pCEP specific (Guiral, 2006) |
| pCEPR | AGGAGACATTCCTTCCGTATC |  |
| specF | ATCGATTTTCGTTCGTGAATACATGTTAT | pDL278 specific (Yesilkaya, 2000) |
| specR | GTTATGCAAGGGTTTATTGTTTTCTA |  |
| ***Cloning for protein expression*** | | |
| tpx-for | CCGACTGTTTCTCGAGAAATGGAAGAAA | TpxD |
| tpx-rev | TGACAGAGGTACCTTTCTATAGGGC |  |
| CodYF | GTATTTTCAGGGCGCCATGGCACATTTATTAGAA | CodY |
| CodYR | GACGGAGCTCGAATTTCGTAATCTCTTTTCTTCACT |  |
| ***EMSA analysis*** | | |
| Spd1464E1FAM | CTCATGTGAGCTGGCGTTT | DNA probe |
| Spd1464E2 | TACCATAGGATACTCCAATC | DNA probe |
| ***Gene expression analysis*** | | |
| tpxRTF | AGAATTGGCTGGACTGGACAA | *tpxD* |
| tpxRTR | CACCGCACCAACGTTTTTG |  |
| gyrARTF | CGTAGAGAATGCGACGGTGAA | *gyrA* |
| gyrARTR | GTTATCGTAGCGCGAGCTCTTC |  |
| brnQRTF | TAGCCCCCTTTTGTCGGATG | *brnQ* |
| brnQRTR | GCTCCCAGAACGACCAAGAT |  |
| spd1652RTF | TCAACCGTACCCTTGCCATC | *SPD1652* |
| spd1652RTR | CGCCTCCGCTTAGATACCAG |  |
| gorRTF | AGTCAGTTCTTTCTCGCCCG | *gor* |
| gorRTR | ACTCTGAACGAACGTGGCTT |  |
| nrdDRTF | CAGGTCGCATGAATCTGGGT | *nrdD* |
| nrdDRTR | GCTGGTGTCGCCTCTTTAGT |  |
| grpERTF | ATATCCAACGCCGTGCCAAT | *grpE* |
| grpERTR | ATCGCCAAGCCCTTCTTCAC |  |
| clpLRTF | GGAGACGCAGGTGTTGGTAA | *clpL* |
| clpLRTR | GGACATCGCCGTTCACAATC |  |
| ilvDRTF | ATGGAGTCCGCGATGATGTC | *ilvD* |
| ilvDRTR | TGATTTCGACTTGGGCGGAA |  |
| codYRTF | GATTGCCAGTACCGTTGT | *codY* |
| codYRTR | CACGGAGTTCGGAGTAAG |  |

^a^ Gene numbers refer to D39 locus tags
