# Supplementary material for: CodY Regulates Thiol Peroxidase Expression as Part of the Pneumococcal Defense Mechanism against H2O2 Stress
Source: Front Cell Infect Microbiol. 2017 May 24;7:210. doi: 10.3389/fcimb.2017.00210 (PMC5443158; doi:10.3389/fcimb.2017.00210)
Supplement: Supplementary file 2 [file Table2.DOCX]

**Table S2. Genes differentially expressed in H_2_O_2_-challenged versus unchallenged D39.** Genes identified by microarray that undergo a significant change (≥1.8 fold up- or down-regulated) in expression when anaerobically grown D39 were exposed to 1 mM H_2_O_2_ compared to unchallenged D39.

| **D39 locus tag^a^** | **TIGR4 locus tag^b^** | **Gene name** | **Gene Product** | **Ratio^c^** | **Bayes. p**^d^ |
| --- | --- | --- | --- | --- | --- |
| ***Up-regulated genes*** | | | | | |
| SPD_0546 | SP0626 | *brnQ* | branched-chain amino acid transport system II carrier protein | 9.5 | 3.75E-13 |
| SPD_1650 | SP1870 | *-* | iron-compound ABC transporter permease | 5.6 | 9.02E-07 |
| SPD_1652 | SP1872 | *-* | iron-compound ABC transporter iron-compound-binding protein | 5.5 | 3.29E-09 |
| SPD_0091 | SP0095 | *-* | hypothetical protein SPD_0091 | 5.5 | 3.40E-12 |
| SPD_1651 | SP1871 | *-* | iron-compound ABC transporter ATP-binding protein | 5.1 | 4.18E-07 |
| SPD_0308 | SP0338 | *clpL* | ATP-dependent Clp protease, ATP-binding subunit | 4.9 | 3.36E-13 |
| SPD_0685 | SP0784 | *gor* | glutathione reductase | 4.9 | 1.16E-11 |
| SPD_0187 | SP0202 | *nrdD* | anaerobic ribonucleoside triphosphate reductase | 4.4 | 4.66E-11 |
| SPD_0459 | SP0516 | *grpE* | heat shock protein GrpE | 4.4 | 5.09E-13 |
| SPD_0460 | SP0517 | *dnaK* | molecular chaperone DnaK | 3.6 | 4.79E-12 |
| SPD_0289 | SP0317 | *eda* | keto-hydroxyglutarate-aldolase/keto-deoxy-phosphogluconate aldolase | 3.5 | 1.71E-09 |
| SPD_0461 | SP0519 | *dnaJ* | chaperone protein DnaJ | 3.5 | 4.74E-12 |
| SPD_0189 | SP0204 | *-* | acetyltransferase, GNAT family protein | 3.5 | 2.56E-10 |
| SPD_0458 | SP0515 | *hrcA* | heat-inducible transcription repressor | 3.3 | 1.53E-10 |
| SPD_0190 | SP0205 | *nrdG* | anaerobic ribonucleoside-triphosphate reductase activating protein | 3.0 | 4.09E-10 |
| SPD_1649 | SP1869 | *-* | iron-compound ABC transporter permease | 2.9 | 9.38E-05 |
| SPD_0104 | SP0107 | *-* | LysM domain-containing protein | 2.8 | 1.58E-08 |
| SPD_0291 | SP0319 | *-* | hypothetical protein SPD_0291 | 2.8 | 4.29E-08 |
| SPD_2033 | SP2206 | *yfiA* | ribosomal subunit interface protein | 2.8 | 7.99E-10 |
| SPD_0290 | SP0318 | *-* | carbohydrate kinase, PfkB family protein | 2.7 | 1.58E-06 |
| SPD_1709 | SP1906 | *groEL* | chaperonin GroEL | 2.7 | 1.41E-07 |
| SPD_1415 | SP1588 | *-* | pyridine nucleotide-disulfide oxidoreductase | 2.6 | 5.28E-06 |
| SPD_0191 | SP0207 | *-* | hypothetical protein SPD_0191 | 2.6 | 6.89E-07 |
| SPD_0357 | SP0391 | *cbpF* | choline binding protein F | 2.5 | 2.37E-09 |
| SPD_0886 | SP1000 | *-* | thioredoxin family protein | 2.5 | 6.67E-07 |
| SPD_0352 | SP0387 | *-* | DNA-binding response regulator | 2.4 | 5.14E-06 |
| SPD_0501 | SP0576 | *licT* | transcription antiterminator Lict | 2.4 | 6.04E-06 |
| SPD_1913 | SP2087 | *pstB* | phosphate ABC transporter ATP-binding protein | 2.2 | 1.28E-06 |
| SPD_1327 | SP1499 | *bta* | bacterocin transport accessory protein | 2.2 | 7.04E-05 |
| SPD_1090 | SP1232 | *-* | hypothetical protein SPD_1090 | 2.2 | 5.85E-08 |
| SPD_0297 | SP0325 | *-* | PTS system, IID component | 2.2 | 8.45E-07 |
| SPD_0350 | SP0385 | *-* | hypothetical protein SPD_0350 | 2.1 | 1.32E-06 |
| SPD_1916 | SP2090 | *-* | transcriptional regulator | 2.1 | 2.43E-05 |
| SPD_1267 | SP1438 | *-* | ABC transporter ATP-binding protein | 2.1 | 0.00028 |
| SPD_0803 | SP0910 | *-* | hypothetical protein SPD_0803 | 2.1 | 6.61E-06 |
| SPD_1089 | SP1231 | *coaC* | phosphopantothenoylcysteine decarboxylase | 2.0 | 2.41E-07 |
| SPD_1251 | SP1421 | *pncB* | nicotinate phosphoribosyltransferase | 2.0 | 0.00045 |
| SPD_1469 | SP1656 | *-* | hypothetical protein SPD_1469 | 2.0 | 0.00055 |
| SPD_0292 | SP0320 | *-* | gluconate 5-dehydrogenase | 2.0 | 3.66E-05 |
| SPD_2069 | SP2240 | *-* | SpoJ protein | 2.0 | 9.78E-09 |
| SPD_2065 | SP2237 | *comC1* | competence-stimulating peptide type 1 | 2.0 | 0.00083 |
| SPD_0805 | SP0913 | *-* | transporter permease, | 2.0 | 0.0004 |
| SPD_1769 | SP1972 | *-* | hypothetical protein SPD_1769 | 2.0 | 8.25E-06 |
| SPD_2043 | SP2216 | *-* | hypothetical protein | 2.0 | 1.73E-08 |
| SPD_0451 | SP23F_2174 | *-* | type I restriction-modification system, S subunit, | 2.0 | 0.00208 |
| SPD_1468 | SP1655 | *gpmA* | phosphoglyceromutase | 2.0 | 7.71E-07 |
| SPD_0344 | SP0376 | *-* | DNA-binding response regulator | 1.9 | 4.10E-08 |
| SPD_0099 | SP0103 | *capD* | capsular polysaccharide biosynthesis protein | 1.9 | 1.59E-07 |
| SPD_1932 | SP2106 | *malP* | maltodextrin phosphorylase | 1.9 | 1.91E-06 |
| SPD_0098 | SP0102 | *-* | glycosyl transferase, group 2 family protein | 1.9 | 3.08E-08 |
| SPD_0351 | SP0386 | *-* | sensor histidine kinase, | 1.9 | 6.19E-06 |
| SPD_1933 | SP2107 | *malQ* | 4-alpha-glucanotransferase | 1.9 | 5.85E-07 |
| SPD_0995 | SP1111 | *-* | hypothetical protein SPD_0995 | 1.9 | 6.55E-06 |
| SPD_1043 | SP1180 | *nrdF* | ribonucleotide-diphosphate reductase subunit beta | 1.9 | 2.45E-08 |
| SPD_1710 | SP1907 | *groES* | co-chaperonin GroES | 1.9 | 0.00070 |
| SPD_0001 | SP0001 | *dnaA* | chromosomal replication initiation protein | 1.8 | 3.91E-07 |
| SPD_0013 | SP0013 | *ftsH* | cell division protein FtsH | 1.8 | 4.06E-07 |
| SPD_1042 | SP1179 | *nrdE* | ribonucleotide-diphosphate reductase subunit alpha | 1.8 | 6.36E-07 |
| SPD_1506 | SP1695 | *-* | acetyl xylan esterase, | 1.8 | 6.96E-05 |
| SPD_1914 | SP2088 | *phoU* | phosphate transport system regulatory protein PhoU | 1.8 | 4.08E-05 |
| SPD_1456 | SP1643 | *-* | hypothetical protein SPD_1456 | 1.8 | 0.0016 |
| SPD_0437 | SP0488 | *-* | hypothetical protein SPD_0437 | 1.8 | 4.15E-07 |
| SPD_1464 | SP1651 | *tpx* | thiol peroxidase | 1.8 | 0.002149601 |
| SPD_0663 | SP0761 | *-* | ATP-dependent RNA helicase, DEAD/DEAH box family protein | 1.8 | 0.00092 |
| SPD_0626 | SP0720 | *-* | ABC transporter ATP-binding protein | 1.8 | 0.00134 |
| SPD_0537 | SP0617 | *-* | hypothetical protein SPD_0537 | 1.8 | 3.93E-08 |
| SPD_2002 | SP2173 | *dltD* | undecaprenol-phosphate-poly(glycerophosphate subunit) D-alanine transfer protein | 1.8 | 2.44E-06 |
| SPD_0420 | SP0459 | *pflB* | formate acetyltransferase | 1.8 | 1.67E-06 |
| SPD_1594 | SP1809 | *-* | transcriptional regulator | 1.8 | 0.00095 |
| SPD_1767 | SP1969 | *-* | type II DNA modification methyltransferase, | 1.8 | 2.45E-05 |
| SPD_0466 | SP0524 | *-* | hypothetical protein SPD_0466 | 1.8 | 0.00187 |
| ***Down-regulated genes*** | | | | | |
| SPD_0334 | SP0366 | *aliA* | oligopeptide ABC transporter oligopeptide-binding protein AliA | -4.4 | 6.04E-12 |
| SPD_1134 | SP1278 | *pyrR* | pyrimidine regulatory protein PyrR | -4.2 | 1.32E-10 |
| SPD_1956 | SP2126 | *ilvD* | dihydroxy-acid dehydratase | -4.2 | 1.33E-13 |
| SPD_1461 | SP1648 | *psaB* | manganese ABC transporter ATP-binding protein | -4.1 | 3.14E-11 |
| SPD_1462 | SPXX37 | *psaC* | manganese ABC transporter permease, | -3.7 | 4.44E-09 |
| SPD_0852 | SP0964 | *pyrDb* | dihydroorotate dehydrogenase 1B | -3.7 | 4.31E-08 |
| SPD_1133 | SP1277 | *pyrB* | aspartate carbamoyltransferase catalytic subunit | -3.5 | 1.66E-12 |
| SPD_0609 | SP0702 | *pyrE* | orotate phosphoribosyltransferase | -3.5 | 8.37E-08 |
| SPD_0382 | SP0419 | *fabK* | trans-2-enoyl-ACP reductase II | -3.5 | 3.61E-10 |
| SPD_0447 | SP0501 | *-* | transcriptional regulator, MerR family protein | -3.3 | 4.58E-11 |
| SPD_1729 | SP1926 | *-* | hypothetical protein SPD_1729 | -3.1 | 3.51E-07 |
| SPD_0263 | SP0283 | *manM* | PTS system, mannose-specific IIC component | -3.1 | 3.81E-10 |
| SPD_1899 | SP2072 | *-* | glutamine amidotransferase, class 1 | -3.1 | 1.94E-11 |
| SPD_1258 | SP1429 | *-* | peptidase, U32 family protein | -3.1 | 2.35E-09 |
| SPD_1000 | SP1116 | *-* | transporter major facilitator family protein | -3.0 | 5.06E-05 |
| SPD_0383 | SP0420 | *fabD* | acyl-carrier-protein S-malonyltransferase | -3.0 | 2.43E-11 |
| SPD_0264 | SP0284 | *manL* | PTS system, mannose-specific IIAB components | -2.8 | 5.81E-11 |
| SPD_1099 | SP1242 | *-* | amino acid ABC transporter ATP-binding protein | -2.8 | 8.37E-10 |
| SPD_0405 | SP0446 | *ilvH* | acetolactate synthase 3 regulatory subunit | -2.8 | 4.94E-06 |
| SPD_0262 | SP0282 | *-* | PTS system, mannose/fructose/sorbose family protein, IID component | -2.8 | 1.18E-09 |
| SPD_0851 | SP0963 | *pyrK* | dihydroorotate dehydrogenase electron transfer subunit | -2.8 | 7.92E-06 |
| SPD_1936 | SP2110 | *malD* | maltodextrin ABC transporter permease | -2.8 | 8.13E-08 |
| SPD_1587 | SP1800 | *-* | transcriptional activator, | -2.7 | 4.20E-06 |
| SPD_1602 | SP1817 | *trpE* | anthranilate synthase component I | -2.7 | 0.00257 |
| SPD_1965 | SP2136 | *pcpA* | choline binding protein PcpA | -2.7 | 3.16E-08 |
| SPD_1413 | SP1586 | *-* | ATP-dependent RNA helicase, | -2.7 | 9.59E-12 |
| SPD_0384 | SP0421 | *fabG* | 3-ketoacyl-(acyl-carrier-protein) reductase | -2.6 | 1.33E-09 |
| SPD_1402 | SP1572 | *-* | non-heme iron-containing ferritin | -2.6 | 2.91E-11 |
| SPD_1864 | SP2054 | *-* | hypothetical protein SPD_1864 | -2.6 | 7.10E-07 |
| SPD_1107 | SP1249 | *guaC* | guanosine 5'-monophosphate oxidoreductase | -2.6 | 1.66E-08 |
| SPD_0373 | SP0409 | *-* | hypothetical protein SPD_0373 | -2.5 | 1.65E-07 |
| SPD_1450 | SP1638 | *psaR* | iron-dependent transcriptional regulator | -2.5 | 5.58E-05 |
| SPD_0668 | SP0767 | *-* | hypothetical protein SPD_0668 | -2.5 | 0.00136 |
| SPD_1935 | SP2109 | *malC* | maltodextrin ABC transporter permease | -2.5 | 3.45E-05 |
| SPD_1151 | SP1296 | *-* | hypothetical protein SPD_1151 | -2.4 | 0.00025 |
| SPD_0608 | SP0701 | *pyrF* | orotidine 5'-phosphate decarboxylase | -2.4 | 4.32E-05 |
| SPD_0214 | SP0231 | *adk* | adenylate kinase | -2.4 | 4.97E-09 |
| SPD_0230 | SP0246 | *-* | transcriptional regulator, DeoR family protein | -2.4 | 2.10E-06 |
| SPD_1800 | SP2002 | *-* | hypothetical protein SPD_1800 | -2.4 | 0.00032 |
| SPD_0404 | SP0445 | *ilvB* | acetolactate synthase catalytic subunit | -2.4 | 1.13E-07 |
| SPD_0409 | SP0450 | *ilvA* | threonine dehydratase | -2.4 | 3.57E-06 |
| SPD_1004 | SP1119 | *gapN* | glyceraldehyde-3-phosphate dehydrogenase, NADP-dependent | -2.4 | 2.54E-05 |
| SPD_0480 | SP0554 | *-* | hypothetical protein SPD_0480 | -2.3 | 9.43E-06 |
| SPD_1158 | SP1306 | *gdhA* | glutamate dehydrogenase | -2.3 | 1.52E-09 |
| SPD_0386 | SP0423 | *accB* | acetyl-CoA carboxylase biotin carboxyl carrier protein subunit | -2.3 | 2.66E-07 |
| SPD_1631 | SP1850 | *-* | Dam-replacing family protein | -2.3 | 4.71E-05 |
| SPD_1726 | SP1923 | *ply* | pneumolysin | -2.3 | 1.84E-06 |
| SPD_1868 | SP2058 | *tgt* | queuine tRNA-ribosyltransferase | -2.3 | 0.00029 |
| SPD_1798 | SP2000 | *-* | DNA-binding response regulator | -2.3 | 8.31E-05 |
| SPD_1727 | SP1924 | *-* | hypothetical protein SPD_1727 | -2.3 | 9.87E-07 |
| SPD_1664 | SP1884 | *-* | PTS system, trehalose-specific IIABC components | -2.3 | 7.07E-05 |
| SPD_1108 | SP1250 | *-* | hypothetical protein SPD_1108 | -2.3 | 0.00072 |
| SPD_1001 | SP1117 | *ligA* | NAD-dependent DNA ligase LigA | -2.3 | 0.00012 |
| SPD_0107 | SP0110 | *-* | membrnae protein, | -2.2 | 4.19E-09 |
| SPD_1306 | SP1476 | *-* | hypothetical protein SPD_1306 | -2.2 | 0.00137 |
| SPD_1132 | SP1276 | *carA* | carbamoyl phosphate synthase small subunit | -2.2 | 2.70E-10 |
| SPD_0686 | SP0785 | *-* | hypothetical protein SPD_0686 | -2.2 | 1.49E-05 |
| SPD_1115 | SPXX35 | *leuB* | 3-isopropylmalate dehydrogenase | -2.2 | 0.00015 |
| SPD_1524 | SP1714 | *-* | transcriptional regulator, GntR family protein | -2.2 | 7.91E-07 |
| SPD_1570 | SP1779 | *-* | hypothetical protein SPD_1570 | -2.2 | 0.00178 |
| SPD_1105 | SP1248 | *rnc* | ribonuclease III | -2.2 | 5.54E-06 |
| SPD_0379 | SP0416 | *-* | transcriptional regulator, MarR family protein | -2.2 | 6.95E-07 |
| SPD_2050 | SP2223 | *-* | hypothetical protein SPD_2050 | -2.2 | 4.14E-09 |
| SPD_1829 | SP2020 | *-* | transcriptional regulator, GntR family protein | -2.2 | 1.75E-05 |
| SPD_0406 | SP0447 | *ilvC* | ketol-acid reductoisomerase | -2.2 | 1.18E-07 |
| SPD_1374 | SP1545 | *-* | hypothetical protein SPD_1374 | -2.2 | 6.37E-07 |
| SPD_0231 | SP0247 | *-* | transcriptional activator | -2.2 | 9.29E-05 |
| SPD_0449 | SP0504 | *-* | hypothetical protein SPD_0449 | -2.2 | 0.00037 |
| SPD_1098 | SP1241 | *-* | amino acid ABC transporter amino acid-binding protein/permease | -2.1 | 1.74E-09 |
| SPD_1954 | SP2125 | *-* | hypothetical protein SPD_1954 | -2.1 | 0.0004 |
| SPD_1007 | SP1123 | *glgD* | glucose-1-phosphate adenylyltransferase, GlgD subunit | -2.1 | 5.34E-05 |
| SPD_0215 | SP0232 | *infA* | translation initiation factor IF-1 | -2.1 | 1.24E-05 |
| SPD_0818 | SP0927 | *-* | transcriptional regulator, LysR family protein | -2.1 | 0.00015 |
| SPD_0005 | SP0005 | *pth* | peptidyl-tRNA hydrolase | -2.1 | 0.00024 |
| SPD_1118 | SP1260 | *cutC* | copper homeostasis protein CutC | -2.1 | 0.00203 |
| SPD_1728 | SP1925 | *-* | hypothetical protein SPD_1728 | -2.1 | 4.95E-06 |
| SPD_0825 | SP0935 | *tmk* | thymidylate kinase | -2.1 | 4.13E-05 |
| SPD_0097 | SP0101 | *-* | transporter | -2.1 | 0.00077 |
| SPD_1997 | SP2169 | *adcA* | zinc ABC transporter zinc-binding lipoprotein | -2.1 | 2.56E-07 |
| SPD_0387 | SP0424 | *fabZ* | (3R)-hydroxymyristoyl-ACP dehydratase | -2.1 | 2.77E-08 |
| SPD_0194 | SP0210 | *rplD* | 50S ribosomal protein L4 | -2.1 | 3.12E-07 |
| SPD_1383 | SP1551 | *-* | cation-transporting ATPase, E1-E2 family protein | -2.1 | 3.65E-06 |
| SPD_0504 | SP0579 | *pheS* | phenylalanyl-tRNA synthetase subunit alpha | -2.0 | 0.00095 |
| SPD_1605 | SP1821 | *-* | LacI family transcriptional regulator | -2.0 | 0.00015 |
| SPD_1008 | SP1124 | *glgA* | glycogen synthase | -2.0 | 0.00025 |
| SPD_0388 | SP0425 | *accC* | acetyl-CoA carboxylase biotin carboxylase subunit | -2.0 | 2.93E-07 |
| SPD_1248 | SPXX64 | *-* | hypothetical protein SPD_1248 | -2.0 | 3.71E-05 |
| SPD_0251 | SP0271 | *rpsL* | 30S ribosomal protein S12 | -2.0 | 0.00013 |
| SPD_0990 | SP1106 | *-* | hypothetical protein SPD_0990 | -2.0 | 0.00015 |
| SPD_0905 | SP1019 | *-* | acetyltransferase, GNAT family protein | -2.0 | 0.00036 |
| SPD_0378 | SP0415 | *-* | enoyl-CoA hydratase | -2.0 | 1.08E-08 |
| SPD_1347 | SP1519 | *-* | acetyltransferase, GNAT family protein | -2.0 | 0.00052 |
| SPD_0757 | SP0862 | *rpsA* | 30S ribosomal protein S1 | -2.0 | 4.94E-06 |
| SPD_1028 | SP1164 | *acoA* | TPP-dependent acetoin dehydrogenase alpha-subunit | -2.0 | 3.24E-06 |
| SPD_1826 | SP2016 | *nadC* | nicotinate-nucleotide pyrophosphorylase | -2.0 | 0.00318 |
| SPD_1222 | SP1390 | *murB* | UDP-N-acetylenolpyruvoylglucosamine reductase | -2.0 | 0.00039 |
| SPD_0202 | SP0218 | *rpsQ* | 30S ribosomal protein S17 | -2.0 | 6.37E-06 |
| SPD_1819 | SP2007 | *nusG* | transcription antitermination protein NusG | -2.0 | 0.00043 |
| SPD_1215 | SP1382 | *amy* | cytoplasmic alpha-amylase | -1.9 | 7.21E-08 |
| SPD_0144 | SP0141 | *-* | transcriptional regulator | -1.9 | 6.25E-05 |
| SPD_1525 | SP1715 | *-* | ABC transporter ATP-binding protein | -1.9 | 5.10E-08 |
| SPD_0635 | SP0729 | *-* | cation-transporting ATPase, E1-E2 family protein | -1.9 | 7.22E-05 |
| SPD_1373 | SP1544 | *aspC* | aspartate aminotransferase | -1.9 | 8.45E-07 |
| SPD_1706 | SP1903 | *-* | hypothetical protein SPD_1706 | -1.9 | 0.00017 |
| SPD_0654 | SP0751 | *livM* | branched-chain amino acid ABC transporter permease | -1.9 | 0.00037 |
| SPD_0204 | SP0220 | *rplX* | 50S ribosomal protein L24 | -1.9 | 1.24E-08 |
| SPD_1937 | SP2111 | *malA* | maltodextrose utilization protein MalA | -1.9 | 0.00025 |
| SPD_0900 | SP1013 | *asd* | aspartate-semialdehyde dehydrogenase | -1.9 | 7.89E-08 |
| SPD_1875 | SP2064 | *-* | hydrolase, haloacid dehalogenase-like family protein | -1.9 | 0.00173 |
| SPD_0850 | SP0962 | *gloA* | lactoylglutathione lyase | -1.9 | 0.00028 |
| SPD_1141 | SP1286 | *uraA* | uracil-xanthine permease | -1.9 | 4.34E-05 |
| SPD_1302 | SP1472 | *-* | oxidoreductase, | -1.9 | 0.00135 |
| SPD_1104 | SP1247 | *smc* | chromosome segregation protein SMC | -1.9 | 1.84E-06 |
| SPD_0003 | SP0003 | *-* | hypothetical protein SPD_0003 | -1.9 | 0.00245 |
| SPD_1597 | SP1812 | *trpB* | tryptophan synthase subunit beta | -1.9 | 0.00124 |
| SPD_1226 | SP1394 | *-* | amino acid ABC transporter amino acid-binding protein | -1.9 | 2.86E-05 |
| SPD_0848 | SP0960 | *rpmI* | 50S ribosomal protein L35 | -1.8 | 0.00126 |
| SPD_2031 | SP2204 | *rplI* | 50S ribosomal protein L9 | -1.8 | 2.97E-07 |
| SPD_0267 | SP0287 | *-* | xanthine/uracil permease family protein | -1.8 | 0.0008 |
| SPD_1217 | SP1384 | *-* | hypothetical protein SPD_1217 | -1.8 | 5.63E-06 |
| SPD_1300 | SP1470 | *-* | thiamine biosynthesis protein ApbE, | -1.8 | 0.00022 |
| SPD_0198 | SP0214 | *rplV* | 50S ribosomal protein L22 | -1.8 | 2.67E-05 |
| SPD_1233 | SP1402 | *-* | NOL1/NOP2/sun family protein | -1.8 | 0.00059 |
| SPD_0009 | SP0009 | *-* | hypothetical protein SPD_0009 | -1.8 | 0.00457 |
| SPD_1671 | SP1891 | *amiA* | oligopeptide ABC transporter oligopeptide-binding protein AmiA | -1.8 | 0.00019 |
| SPD_1598 | SP1813 | *trpF* | N-(5'-phosphoribosyl)anthranilate isomerase | -1.8 | 0.00218 |
| SPD_1024 | SP1160 | *-* | lipoate-protein ligase, | -1.8 | 0.00067 |
| SPD_0970 | SP1084 | *map* | methionine aminopeptidase | -1.8 | 0.00157 |
| SPD_0901 | SP1014 | *dapA* | dihydrodipicolinate synthase | -1.8 | 0.0010 |
| SPD_1329 | SP1501 | *-* | amino acid ABC transporter ATP-binding protein | -1.8 | 0.00215 |
| SPD_0588 | SP0676 | *-* | transcriptional regulator, | -1.8 | 0.0028 |
| SPD_1412 | SP1584 | *codY* | transcriptional repressor CodY | -1.8 | 0.00015 |
| SPD_0962 | SP1077 | *-* | hypothetical protein SPD_0962 | -1.8 | 5.08E-05 |
| SPD_1234 | SP1403 | *-* | inositol monophosphatase family protein | -1.8 | 0.00385 |
| SPD_0094 | SP0098 | *-* | hypothetical protein SPD_0094 | -1.8 | 1.79E-07 |
| SPD_0801 | SP0907 | *-* | hypothetical protein SPD_0801 | -1.8 | 0.00030 |
| SPD_0890 | SP1004 | *phtE* | pneumococcal histidine triad protein E precursor | -1.8 | 1.10E-05 |
| SPD_0967 | SP1081 | *murA-1* | UDP-N-acetylglucosamine 1-carboxyvinyltransferase | -1.8 | 0.00018 |
| SPD_0494 | SP0568 | *valS* | valyl-tRNA synthetase | -1.8 | 0.00227 |
| SPD_1493 | SP1681 | *-* | sugar ABC transporter permease | -1.8 | 0.0049 |
| SPD_0655 | SP0752 | *livG* | branched-chain amino acid ABC transporter ATP-binding protein | -1.8 | 0.00072 |
| SPD_2014 | SP2187 | *-* | hypothetical protein SPD_2014 | -1.8 | 0.00068 |
| SPD_1876 | SP2065 | *-* | MATE efflux family protein | -1.8 | 0.00202 |
| SPD_0880 | SP0994 | *-* | hypothetical protein SPD_0880 | -1.8 | 0.0026 |
| SPD_1378 | SP1547 | *-* | hypothetical protein SPD_1378 | -1.8 | 6.62E-05 |
| SPD_1508 | SP1698 | *alr* | alanine racemase | -1.8 | 0.00060 |

^a^ Gene numbers refer to D39 locus tags**;** ^b^ Gene numbers refer to TIGR4 locus tags; ^c^ Ratio ≥1.8 or ≤-1.8 (D39 challenged with 1 mM H_2_O_2_ compared to unchallenged D39); ^d^ Bayesian p value.
