# Supplementary material for: CodY Regulates Thiol Peroxidase Expression as Part of the Pneumococcal Defense Mechanism against H2O2 Stress
Source: Front Cell Infect Microbiol. 2017 May 24;7:210. doi: 10.3389/fcimb.2017.00210 (PMC5443158; doi:10.3389/fcimb.2017.00210)
Supplement: Supplementary file 3 [file Table3.DOCX]

**Table S3.** **Genes differentially expressed in H_2_O_2_-challenged versus unchallenged Δ*tpxD* mutant.** Genes identified by microarray that undergo a significant change in expression when anaerobically grown Δ*tpxD* mutant cells were exposed to 1 mM H_2_O_2_ compared to unchallenged Δ*tpxD* mutant.

| **D39 locus**  **tag^a^** | **TIGR4**  **locus tag^b^** | **Gene**  **name** | **Gene Product** | **Ratio^c^** | **Bayes. p**^d^ |
| --- | --- | --- | --- | --- | --- |
| ***Up-regulated genes*** | | | | | |
| SPD_0649 | SP0745 | *upp* | uracil phosphoribosyltransferase | 2.3 | 3.11E-03 |
| _*_ | SP0487 | *-* | uncharacterized protein | 1.7 | 1.93E-03 |
| SPD_1933 | SP2107 | *malQ* | 4-alpha-glucanotransferase | 1.5 | 8.80E-03 |
| _*_ | SP1103 | *-* | uncharacterized protein | 1.5 | 3.87E-03 |
| SPD_0013 | SP0013 | *ftsH* | cell division protein FtsH | 1.5 | 2.11E-03 |
| SPD_1253 | SP1423 | *-* | transcriptional repressor, | 1.5 | 9.86E-03 |
| SPD_1530 | SP1720 | *-* | hypothetical protein SPD_1530 | 1.4 | 3.85E-03 |
| SPD_2025 | SP2197 | *-* | ABC transporter substrate-binding protein | 1.4 | 6.65E-03 |
| ***Down-regulated genes*** | | | | | |
| SPD_0126 | SP0117 | *pspA* | pneumococcal surface protein A | -1.5 | 5.03E-03 |
| SPD_1105 | SP1248 | *rnc* | ribonuclease III | -1.5 | 5.56E-03 |
| SPD_0231 | SP0247 | *-* | transcriptional activator | -1.6 | 3.00E-03 |

^a^ Gene numbers refer to D39 locus tags**;** ^b^ Gene numbers refer to TIGR4 locus tags; ^c^ Ratio ≥1.4 or ≤-1.4 (Δ*tpxD* mutant challenged with 1mM H_2_O_2_ compared to unchallenged Δ*tpxD* mutant); ^d^ Bayesian p value.

* Not annotated in NCBI database, despite 99-100% DNA sequence identity to TIGR4 genome.
