# Supplementary material for: CodY Regulates Thiol Peroxidase Expression as Part of the Pneumococcal Defense Mechanism against H2O2 Stress
Source: Front Cell Infect Microbiol. 2017 May 24;7:210. doi: 10.3389/fcimb.2017.00210 (PMC5443158; doi:10.3389/fcimb.2017.00210)
Supplement: Supplementary file 4 [file Table4.DOCX]

**Table S4**. **Number of genes significantly affected by 1 mM H_2_O_2_ in D39 and ∆*tpxD* mutant, divided into functional categories.**

|  | **Functional categories** | **D39** | | | **∆*tpxD*** | | |
| --- | --- | --- | --- | --- | --- | --- | --- |
|  |  | **Total** | **Down** | **Up** | **Total** | **Down** | **Up** |
| [E] | Amino acid transport and metabolism | 26 | 24 | 2 | 0 | 0 | 0 |
| [K] | Transcription | 22 | 16 | 6 | 0 | 0 | 0 |
| [J] | Translation, ribosomal structure and biogenesis | 21 | 19 | 2 | 0 | 0 | 0 |
| [G] | Carbohydrate transport and metabolism | 20 | 12 | 8 | 1 | 0 | 1 |
| [R] | General function prediction only | 18 | 10 | 8 | 1 | 1 | 0 |
| [P] | Inorganic ion transport and metabolism | 14 | 7 | 7 | 1 | 0 | 1 |
| [F] | Nucleotide transport and metabolism | 13 | 9 | 4 | 1 | 0 | 1 |
| [O] | Posttranslational modification, protein turnover, chaperones | 11 | 1 | 10 | 1 | 0 | 1 |
| [C] | Energy production and conversion | 9 | 5 | 4 | 0 | 0 | 0 |
| [I] | Lipid transport and metabolism | 7 | 6 | 1 | 0 | 0 | 0 |
| [M] | Cell wall/membrane/envelope biogenesis | 7 | 5 | 2 | 0 | 0 | 0 |
| [V] | Defense mechanisms | 6 | 5 | 1 | 0 | 0 | 0 |
| [H] | Coenzyme transport and metabolism | 5 | 3 | 2 | 3 | 2 | 1 |
| [L] | Replication, recombination and repair | 3 | 1 | 2 | 0 | 0 | 0 |
| [D] | Cell cycle control, cell division, chromosome partitioning | 1 | 1 | 0 | 0 | 0 | 0 |
| [Q] | Secondary metabolites biosynthesis, transport and catabolism | 1 | 0 | 1 | 0 | 0 | 0 |
| [T] | Signal transduction mechanisms | 1 | 0 | 1 | 0 | 0 | 0 |
| [S] | Function unknown | 32 | 22 | 10 | 1 | 0 | 1 |
|  | **Total number of genes** | **217** | **146** | **71** | **9** | **3** | **6** |
