# Supplementary material for: CodY Regulates Thiol Peroxidase Expression as Part of the Pneumococcal Defense Mechanism against H2O2 Stress
Source: Front Cell Infect Microbiol. 2017 May 24;7:210. doi: 10.3389/fcimb.2017.00210 (PMC5443158; doi:10.3389/fcimb.2017.00210)
Supplement: Supplementary file 5 [file Table5.DOCX]

**Table S5. Genes regulated by CodY that were affected by 1 mM H_2_O_2_, by at least a factor of 1.8.**

| **D39 locus tag^a^** | **Gene name** | **Gene Product** | **Ratio^b^** | **Bayes. p^c^** |
| --- | --- | --- | --- | --- |
| SPD_1956 | *ilvD* | dihydroxy-acid dehydratase | -4.2 | 1.33E-13 |
| SPD_1258 | *-* | peptidase, U32 family protein | -3.1 | 2.35E-09 |
| SPD_0405 | *ilvH* | acetolactate synthase 3 regulatory subunit | -2.8 | 4.94E-06 |
| SPD_1965 | *pcpA* | choline binding protein PcpA | -2.7 | 3.16E-08 |
| SPD_1402 | *-* | non-heme iron-containing ferritin | -2.6 | 2.91E-11 |
| SPD_1004 | *gapN* | glyceraldehyde-3-phosphate dehydrogenase, NADP-dependent | -2.4 | 2.54E-05 |
| SPD_0404 | *ilvB* | acetolactate synthase catalytic subunit | -2.4 | 1.13E-07 |
| SPD_0409 | *ilvA* | threonine dehydratase | -2.4 | 3.57E-06 |
| SPD_1158 | *gdhA* | glutamate dehydrogenase | -2.3 | 1.52E-09 |
| SPD_0406 | *ilvC* | ketol-acid reductoisomerase | -2.2 | 1.18E-07 |
| SPD_1954 | *-* | hypothetical protein SPD_1954 | -2.1 | 4.07E-04 |
| SPD_0900 | *asd* | aspartate-semialdehyde dehydrogenase | -1.9 | 7.89E-08 |
| SPD_0654 | *livM* | branched-chain amino acid ABC transporter permease | -1.9 | 0.000369 |
| SPD_0901 | *dapA* | dihydrodipicolinate synthase | -1.8 | 0.000978 |
| SPD_1671 | *amiA* | oligopeptide ABC transporter oligopeptide-binding protein AmiA | -1.8 | 0.000187 |
| SPD_1412 | *codY* | transcriptional repressor CodY | -1.8 | 0.000147 |
| SPD_0655 | *livG* | branched-chain amino acid ABC transporter ATP-binding protein | -1.8 | 0.000726 |
| SPD_1464 | *tpx* | thiol peroxidase | 1.8 | 0.00215 |
| SPD_1960 | *-* | PTS system, IIB component | 1.9 | 0.008491 |
| SPD_1649 | *-* | iron-compound ABC transporter permease | 2.9 | 9.38E-05 |
| SPD_0459 | *grpE* | heat shock protein GrpE | 4.4 | 5.09E-13 |

^a^ Gene number refer to D39 locus tags**;** ^b^ Ratio ≥1.8 or ≤-1.8 (D39 challenged with 1 mM H_2_O_2_ compared to unchallenged D39); ^c^ Bayesian p value.
