# Supplementary figures and images for: CodY Regulates Thiol Peroxidase Expression as Part of the Pneumococcal Defense Mechanism against H2O2 Stress
Source: Front Cell Infect Microbiol. 2017 May 24;7:210. doi: 10.3389/fcimb.2017.00210 (PMC5443158; doi:10.3389/fcimb.2017.00210)

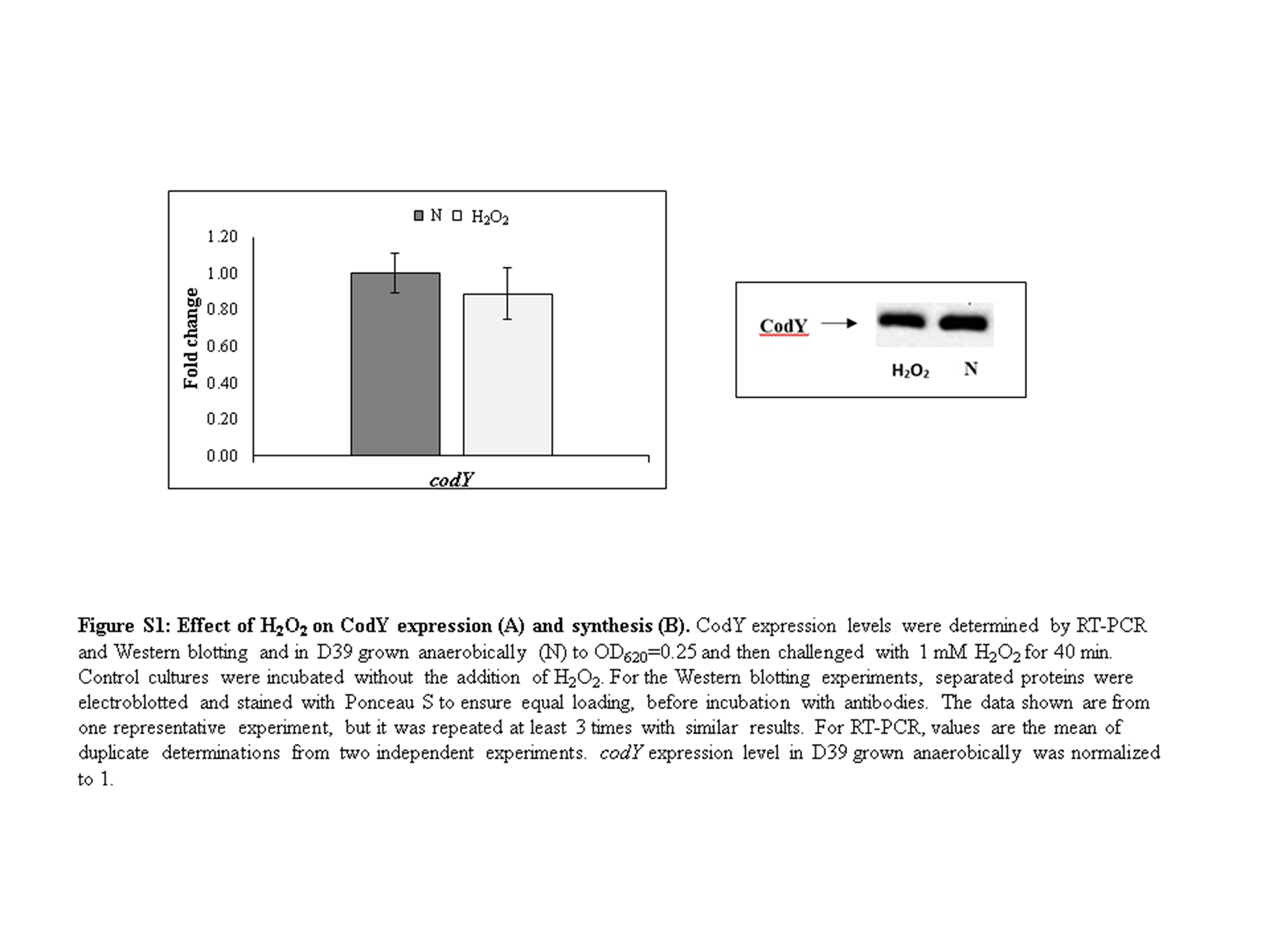

Supplement: Supplementary file 6 [file Image1.TIF]
